# Supplementary material for: Pathfinding quantum simulations of neutrinoless double-β decay
Source: Nat Commun. 2026 Jan 23;17:1826. doi: 10.1038/s41467-026-68536-8 (PMC12921043; doi:10.1038/s41467-026-68536-8)
Supplement: Supplementary file 1 — Supplementary Information [file 41467_2026_68536_MOESM1_ESM.pdf]

Supplementary Information

Supplementary Note 1. Some Relevant Background about $0\nu\beta\beta$-Decay

In the Standard Model, fundamental symmetries prevent neutrinos from having a mass. There is a tremendous amount of evidence that neutrinos are massive [1], and an explanation requires physics beyond the minimal Standard Model. One solution is that neutrinos are their own anti-particles [2, 3], allowing them to receive a Majorana mass contribution at the expense of violating lepton number.[1] A Majorana mass emerges naturally by considering the Standard Model as an effective description of nature that is valid below some high-energy scale. One observable consequence of such a mass term is the $0\nu\beta\beta$-decay of certain nuclei [4]. While $\beta\beta$-decays including the emission of two neutrinos ($2\nu\beta\beta$) proceed via a well-known (doubly-weak) mechanism that conserves lepton number, $0\nu\beta\beta$-decays are forbidden in the minimal Standard Model by lepton-number conservation. This has motivated many ongoing and planned experimental programs that search for signatures of $0\nu\beta\beta$ decay [5–17]. One should keep in mind that a Majorana neutrino mass is not the only mechanism that can give rise to $0\nu\beta\beta$-decay. Contact interactions of higher dimension will generally be present if lepton number is violated, also contributing to the decay rate, e.g., Refs. [18–20]. In either situation, low-energy physics may provide a key window into physics at very high energies.

The $0\nu\beta\beta$-decay rates of nuclei are notoriously difficult to compute, limiting interpretations of lifetime lower bounds predicted by theory and the implications of future potential experimental observations (for an early review, see Ref. [21]). The reasons for this difficulty range from the many-body contributions to matrix elements, for example, as encapsulated in nuclear effective field theory (EFT) treatments (e.g., see Refs. [19, 22–27]), to the coherent evolution of excited states of the nucleus. There have been recent theoretical advances in better understanding the Majorana neutrino decay mechanism in the context of Euclidean-space lattice QCD (e.g., Refs. [23, 28–35]), nuclear shell model (e.g., Refs. [36–40]), and EFT. Specifically, the identification of KSW-type [41–43] logarithms from neutrino exchange [25, 26], leading to the promotion of multi-nucleon contact interactions in the EFT power-counting from renormalization-group scaling.

To better understand aspects of these challenges, it is helpful to dissect the Majorana-mass induced decay mechanism. In the parent nucleus, one time-ordered pathway is that one neutron undergoes single-β decay emitting an e^- and a $\overline{\nu}_e$, transforming into a proton. The $\overline{\nu}_e$ converts into a ν_e via the Majorana mass term, which induces an inverse β-decay of another neutron. The (effective) two-body $nn \to ppe^-e^-$ subprocess occurs within a nucleus, and the amplitudes of all such time-ordered pathways must be summed over. Part of the complexities of this process come from the fact that nucleons in the nucleus are spatially overlapping, strongly interacting and in a correlated two-species fermion wavefunction. At a fundamental level, the β-decay processes are defined in terms of interactions with the quarks via the charged-current weak interaction. These quark-level interactions are evaluated in the nuclear wavefunction and are highly impacted by the correlations between nucleons. In the time-ordered pathway described above, the nucleus time-evolves from the ground state of the strong-interaction Hamiltonian for N neutrons and Z protons, to an intermediate state of the $(N-1, Z+1)$ Hamiltonian, to the ground state of the $(N-2, Z+2)$. In the intermediate time region there are interferences between multiple nuclear energy levels that are coupled with the electroweak Hamiltonian. In the interval of time between the first and second β-decay, the neutrino can be assumed to be freely propagating. However, it is the complete dynamics of the coupled system that is challenging, and whose simulation is potentially better suited for quantum computers.

Supplementary Note 2. Simulation Errors from Approximations

To reduce the depth of quantum circuits, the following approximations were implemented:

- The initial $|\Delta^-\Delta^-\rangle$ states were prepared approximately using SC-ADAPT-VQE. Details are given in Supplementary Note 10, and Supplementary Table 4 shows that the fidelity of the prepared state is 0.99992.
- The range of the chromoelectric interactions is truncated to $\lambda = 1$ staggered site. This is discussed in Supplementary Note 3.
- Time evolution is implemented with $n_T = 2$ first-order Trotter steps.

[1] On general grounds, any lepton number-violating interactions will give rise to a Majorana mass through quantum fluctuations.

- The first Trotter step is simplified using the fact that $|\psi_{\text{vac}}^{(\text{lep})}\rangle|\Delta^-\Delta^-\rangle$ is an eigenstate of the Hamiltonian without $\hat{H}_\beta^{1+1}$. This will be discussed below.

- Pauli terms in the Hamiltonian with coefficients smaller than $t/(16n_T)$ are removed from the time evolution unitary. This effectively removes single-qubit gates with small rotation angles. For the parameters chosen, this simplification only affects terms in the $\hat{H}_{\text{glue}}$ of Supplementary Eq. (10c).

- An approximate $\hat{H}_\beta^{1+1}$ interaction that only acts on the valence quarks is used. In our case, the β-decay process transforms $d \to ue\overline{\nu}$, therefore the terms acting on these fermions are labeled as valence terms, the rest are labeled as sea terms. Analogous to Ref. [44], we approximate the interaction by only keeping the term that acts on the valence space, shown in Supplementary Eq. (8). This corresponds to the first term (and its hermitian conjugate) in Supplementary Eq. (10d).

This supplementary note will compare the exact results obtained from exact diagonalization to results with different levels of approximation.

A. Exact Diagonalization

Simulations of $e^{-i\hat{H}t}|\psi_{\text{init}}\rangle$ using exact diagonalization provide the exact result used to benchmark the impact of the various approximations. Exact diagonalization can be performed on fairly large systems by offloading some of the computation to the tensor product basis, and only constructing explicit matrices when necessary. Enforcing symmetries at the level of the allowed basis states results in a much smaller effective Hilbert space.

As an example, consider a single flavor of quark in 1+1D QCD. In the computational basis, each state is expressed as a string of $3N$ 0s and 1s, giving 2^{3N} states in total. Baryon number $\mathcal{B}$ separately constrains the occupation of the red (r), green (g) and blue (b) fermion sites to satisfy $r = g = b = \mathcal{B}$. This reduces the size of the Hilbert space to $\binom{N}{N/2-\mathcal{B}}^3$ states. In addition, there are also spacetime symmetries and the remaining global $SU(3)$ (color singlet) constraints. Consider the vacuum sector, which has momentum $\mathbf{k} = 0$ and is even under charge conjugation ($C = +1$) and parity ($P = +1$). These symmetries imply that certain bit strings will always contribute to the wavefunctions with equal amplitudes, and with relative signs that can be determined from how the symmetries are realized. The JW mapping makes the realization of symmetries subtle and is discussed in Ref. [45]. In addition, all physical states $|\psi\rangle$ are color singlets, i.e., they satisfy $\sum_n \hat{Q}_n^{(a)}|\psi\rangle = 0$. Out of the eight charges $\hat{Q}^{(a)}$, the two diagonal constraints have already been enforced by selecting $r = g = b = \mathcal{B}$, leaving six additional constraints. One implication of being a color singlet is that, for example, global $SU(3)$ rotations of the form $\exp\left\{i\frac{\pi}{2}\sum_n \hat{Q}_n^{(1)}\right\}|\psi\rangle = |\psi\rangle$ leave the state invariant. This transformation exchanges red and green quarks $r \leftrightarrow g$, while introducing a factor of "i". These constraints, and the corresponding ones for $r \leftrightarrow b$ and $g \leftrightarrow b$, allow for larger sets of bit strings related by symmetry to be grouped together.[2] The remaining non-Abelian constraints are difficult to enforce in the tensor product basis, and the complete color singlet basis can be found from determining the null space of the operator, $\hat{H}_{\mathbf{1}} = (\sum_n Q_n^{(a)})^2$. An efficient way to determine the null space is with the QR factorization of $\hat{H}_{\mathbf{1}}$.

By building the Hamiltonian matrix in a basis that satisfies these constraints, operations such as diagonalization and matrix exponentiation become less computationally demanding. To illustrate its utility, the size of the Hilbert space at different stages in the symmetry projection for the $L = 5$ vacuum sector is

$$2^{30} \xrightarrow{r=g=b=0} 2^{24} \xrightarrow[r\leftrightarrow g,\, g\leftrightarrow b]{\mathbf{k}=0,\, C=P=+1} 2^{18} \xrightarrow{\hat{H}_{\mathbf{1}}|\psi\rangle=0} 2^{15}\ , \tag{1}$$

rounding to the nearest power of 2.

B. Trotterized Time Evolution

The circuits presented in Supplementary Note 10 act on 32 qubits, and can be simulated exactly with a state-vector simulator on a small cluster. The NVIDIA package CUDA-Q [46], running on four A100 GPUs, was used to perform

[2] These constraints do not commute, and in practice only enforcing, e.g., $r \leftrightarrow g$ and $g \leftrightarrow b$ symmetry leads to the smallest basis.

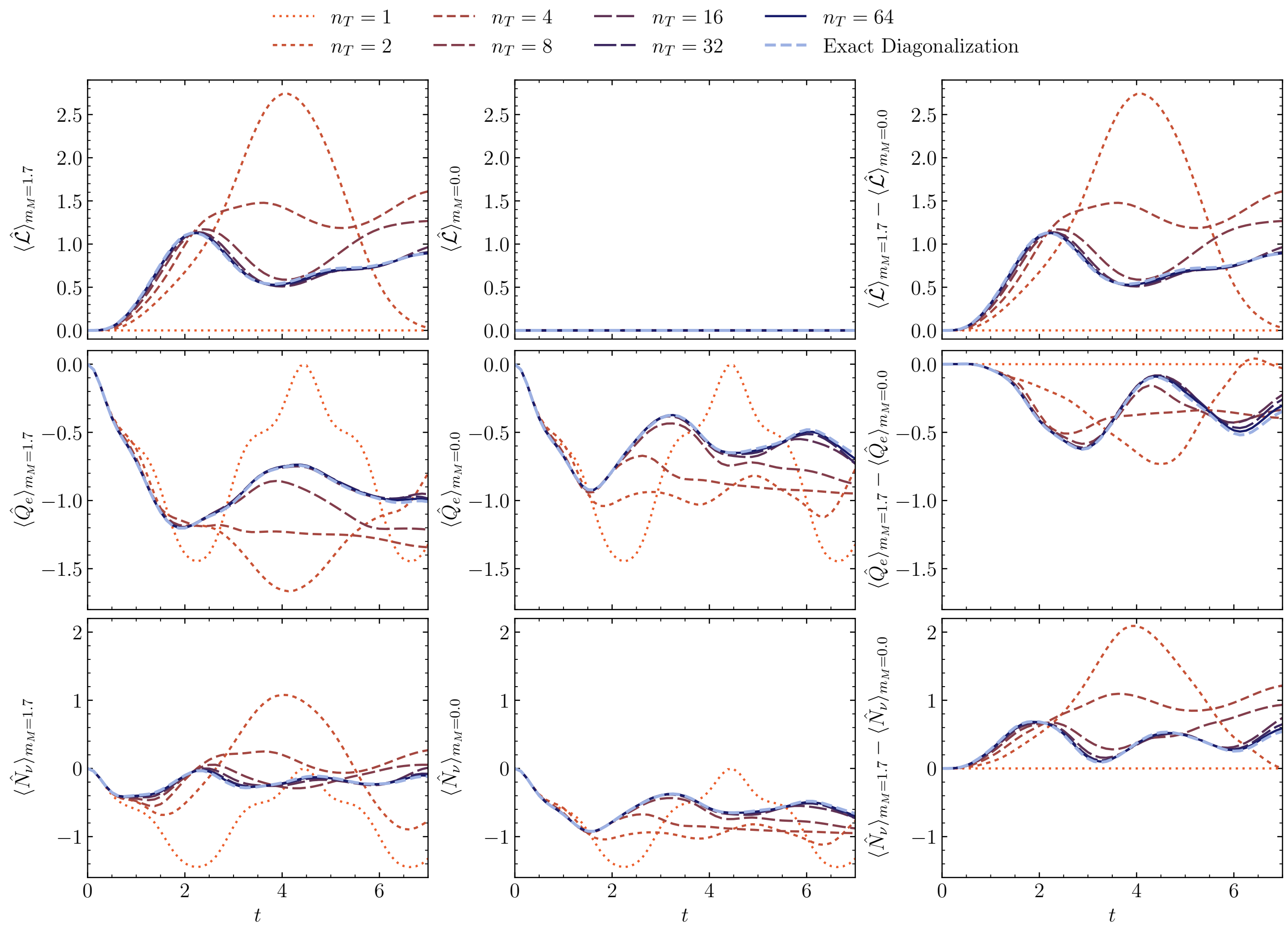

Supplementary Figure 1. The lepton number, $\hat{\mathcal{L}}$, the electric charge in the lepton sector, $\hat{Q}_e$, and the neutrino number, $\hat{N}_\nu$, computed throughout the time evolution starting from $|\psi_{\rm init}\rangle = |\psi_{\rm vac}^{(\rm lep)}\rangle|\Delta^-\Delta^-\rangle$ as a function of the number of Trotter steps. These quantities are computed for $L = 2$ and for two Majorana masses, $m_M = \{0.0, 1.7\}$. No approximations are made in the exact diagonalization results. An exact state-vector simulator is used to compute the time-evolution with n_T Trotter steps, which have Trotter errors, as well as (small) errors coming from the SC-ADAPT-VQE preparation of $|\Delta^-\Delta^-\rangle$. The quantum simulations that we performed on IonQ's Forte-generation quantum processors employed $n_T = 2$ and were limited to $t \leq 2$.

these simulations, together with Qiskit [47] to define the circuits. As noted in our previous work [44], the terms in each Trotter step can be ordered so that the first step has the QCD Hamiltonian acting on an eigenstate. This furnishes an overall phase, and therefore the first Trotter step need only have the term with $\hat{H}_\beta^{1+1}$. Explicitly, for n_T Trotter steps in a leading-order (first-order) Trotter expansion,[3]

$$\begin{aligned} e^{-i\hat{H}t}|\psi_{\rm init}\rangle &\approx \left(e^{-it/n_T \hat{H}_\beta^{1+1}} e^{-it/n_T \hat{H}_{\rm glue}} e^{-it/n_T \hat{H}_{\rm Maj}} e^{-it/n_T \hat{H}_{\rm free}}\right)^{n_T} |\psi_{\rm init}\rangle \\ &\approx \left(e^{-it/n_T \hat{H}_\beta^{1+1}} e^{-it/n_T \hat{H}_{\rm glue}} e^{-it/n_T \hat{H}_{\rm Maj}} e^{-it/n_T \hat{H}_{\rm free}}\right)^{n_T - 1} e^{-it/n_T \hat{H}_\beta^{1+1}} |\psi_{\rm init}\rangle \ , \end{aligned} \tag{2}$$

with $|\psi_{\rm init}\rangle = |\psi_{\rm vac}^{(\rm lep)}\rangle|\Delta^-\Delta^-\rangle$. In this work, the number of Trotter steps n_T is fixed for all the times t. The quantum simulations run on the IonQ Forte-generation QPUs used $n_T = 2$. To quantify the Trotter errors, a comparison between the results from exact diagonalization and $n_T \in \{1, \ldots, 64\}$ is shown in Supplementary Fig. 1, for the lepton

[3] More explicitly, the kinetic terms are broken up into even-odd staggered site and odd-even staggered site hoppings, and the free Hamiltonians are ordered mass then kinetic.

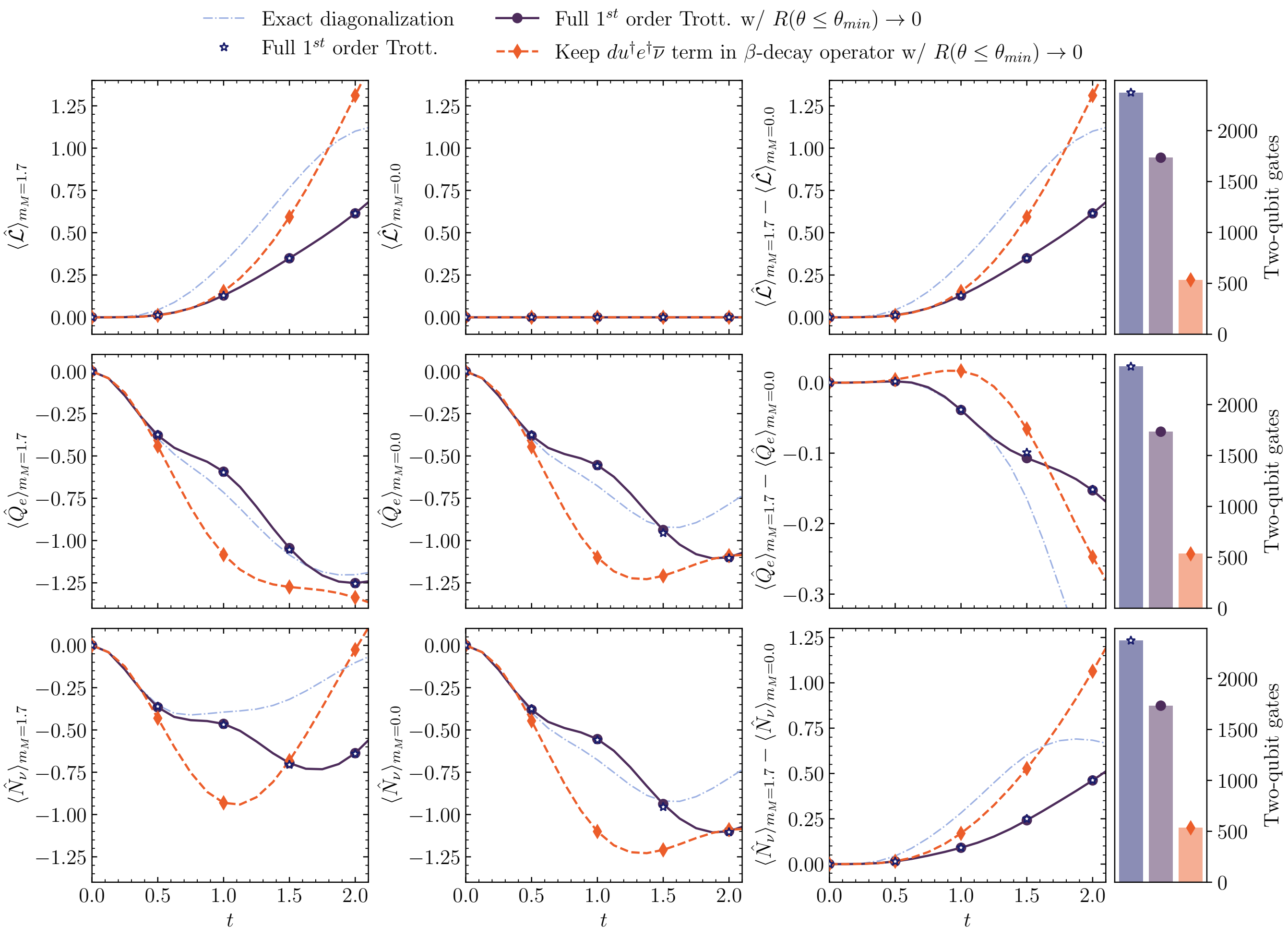

Supplementary Figure 2. The time-evolution of the lepton number, electric charge and neutrino number with varying levels of approximation, as explained in the text. The bar charts to the right give the number of the CZ and CNOT gates required for each level of approximation. The number of two-qubit gates is the same for both the upper and lower panels, and the approximate time evolution is computed using $n_T = 2$ steps of 1st order Trotterization. The results from full 1st order Trotterization and from the angle-truncated implementation essentially coincide.

number, $\hat{\mathcal{L}}$, and the electric charge in the lepton sector, $\hat{Q}_e$, defined in Eq. 1 in the main text. For $t \leq 2.0$, $n_T = 2$ is seen to be reasonably close to the exact result. This justifies our choice of $n_T = 2$ in Results. The maximum number of Trotter steps used, $n_T = 64$, is well converged to the results from exact diagonalization up to $t = 7$.

Note that the expected time evolution does not exhibit the well-known long-time exponential-decay behavior associated with radioactive decay. These deviations were discussed in detail in Ref. [44], and can be attributed to the low-density of final states in a small spatial volume(s). Simulations of these processes performed in increasing volumes should show convergence to the expected exponential behavior.

C. Additional Approximations

To study how the approximations impact our simulations, we study several levels of approximations:

i) Exact diagonalization: the initial state preparation and time evolution are performed exactly.

ii) Full 1st order Trotter: $|\Delta^-\Delta^-\rangle$ is approximately prepared with SC-ADAPT-VQE, the range of the chromoelectric interaction is truncated to $\lambda = 1$ staggered sites and $n_T = 2$ steps of 1st order Trotterized time evolution are used.

Approximation type	Pre-compilation	Post-compilation	Post-compilation + symmetry checks
(iv)	534	454	470
(ii)	2,374	2,292	2,356

Supplementary Table 1. Two-qubit gate counts for circuits executed on IonQ Forte (approximation (ii)) and IonQ Forte Enterprise (approximation (iv)). The gate counts from pre-compilation, post-compilation and post-compilation with additional gates for symmetry checks are shown.

iii) Full 1st order Trotter with $R(\theta \leq \theta_{\text{min}}) \to 0$: The above approximations plus small rotation angles in the Trotterized time evolution circuits set to zero.

iv) Keep $du^\dagger e^\dagger \overline{\nu}$ term: The above approximations plus keeping only the term in $\hat{H}_\beta^{1+1}$ that acts on the valence quarks and valence leptons, the first term in Supplementary Eq. (10d).

The expectations values of lepton number and lepton electric charge with these approximations are shown in Supplementary Fig. 2. The bar charts to the right show the number of two-qubit gates (compiled to CZ and CNOT gates) for each one of these approximations. Approximation (iv) was used in the experiments performed on IonQ Forte Enterprise, and the IonQ circuit compiler reduced the two-qubit gate count from 534 to 470. In particular, circuits were optimized from 534 down to 454 two-qubit gates, and then inflated to 470 after adding mid-circuit symmetry checks (8 leakage checks were added, totaling 16 extra two-qubit gates). For the experiments run on IonQ Forte, approximation (ii) was used, and the IonQ circuit compiler reduced the two-qubit gate count from from 2,374 to 2,356 (2,292 two-qubit gates from the time-evolution circuit, plus 64 additional gates from 4 iSWAP checks). The pre-compilation and post-compilation two-qubit gate counts for approximation (ii) and (iv) are given Supplementary Table 1.

Supplementary Note 3. Additional Details about the Simulation Hamiltonian

This work considers 1+1D QCD with periodic boundary conditions (PBCs) and two flavors of quarks, up u and down d. Discretized onto a staggered lattice with L spatial sites, corresponding to $N = 2L$ staggered sites, the Kogut-Susskind Hamiltonian is [48, 49],

$$\hat{H}_{\text{KS}} = \sum_{f=u,d} \left[\frac{1}{2} \sum_{n=0}^{N-1} \left(\phi_n^{(f)\dagger} U_n \phi_{n+1}^{(f)} + \text{h.c.} \right) + m_f \sum_{n=0}^{N-1} (-1)^n \phi_n^{(f)\dagger} \phi_n^{(f)} \right] + \frac{g^2}{2} \sum_{n=0}^{N-1} \sum_{a=1}^{8} |\mathbf{E}_n^{(a)}|^2 , \tag{3}$$

where $f = \{u, d\}$ labels the quark flavor, m_f is the quark mass and g is the gauge coupling. The quark field operators $\phi_n^{(f)}$ are in the fundamental representation $\mathbf{3}$ of $SU(3)$, and the three color components $\{r, g, b\}$ have been suppressed for simplicity. The chromoelectric field $\mathbf{E}_n^{(a)}$ is in the adjoint representation of $SU(3)$ with $a = \{1, 2, \ldots, 8\}$. The spatial gauge link in $A_t^{(a)} = 0$ (Weyl) gauge is U_n. Staggered sites related by one period are identified, e.g., $\phi_n^{(f)} = \phi_{n+N}^{(f)}$.

Physical states must satisfy Gauss's law, $\mathbf{E}_n^{(a)} - \mathbf{E}_{n-1}^{(a)} = Q_n^{(a)}$, where $Q_n^{(a)} = \sum_f \phi^{(f)\dagger} T^{(a)} \phi^{(f)}$ are the $SU(3)$ charges and the $T^{(a)}$ are the generators of $SU(3)$ in the $\mathbf{3}$ representation, i.e., they are 3×3 matrices. With open boundary conditions, these constraints uniquely determine the state of the the gauge field given the state of the fermions. As a result, explicit gauge field degrees of freedom can be removed, leaving a system of fermions interacting through a Coulomb interaction. With PBCs, there is one spatial mode of the gauge field that is not constrained. In order to keep the translational symmetry manifest, it is beneficial to isolate the $k = 0$ (zero mode) component of the gauge field [50],

$$\begin{aligned} \hat{H}_{\text{KS}} \to & \sum_{f=u,d} \left[\frac{1}{2} \sum_{n=0}^{N-1} \left(\phi_n^{(f)\dagger} U \, \phi_{n+1}^{(f)} + \text{h.c.} \right) + m_f \sum_{n=0}^{N-1} (-1)^n \phi_n^{(f)\dagger} \phi_n^{(f)} \right] \\ & + \frac{N g^2}{2} \sum_{a=1}^{8} \left(E^{(a)} \right)^2 + \frac{g^2}{2} \sum_{s=1}^{L} \left(-s + \frac{s^2}{N} \right) (1 - \frac{1}{2} \delta_{s,L}) \left(\sum_{n=0}^{N-1} \sum_{a=1}^{8} Q_n^{(a)} Q_{n+s}^{(a)} \right) . \end{aligned} \tag{4}$$

Here, $E^{(a)} = \sum_{n=0}^{N-1} E_n^{(a)}/N$ is the zero mode of the electric field, and $U^N = \prod_n U_n$ is its conjugate variable. The gauge field populates a bosonic Hilbert space that is formally infinite dimensional. However, it is expected that

observables will converge rapidly in the chromoelectric field basis if an increasing number of states are kept. In this paper, the gauge-field dynamics are frozen, leaving only fermionic degrees of freedom. The convergence of observables with increasing number of gauge-field states will be quantified in future work. The Hamiltonian that we implement only has fermionic operators,

$$\hat{H}_{\mathrm{KS}} \;\to\; \sum_{f=u,d}\left[\frac{1}{2}\sum_{n=0}^{N-1}\left(\phi_n^{(f)\dagger}\phi_{n+1}^{(f)} + \mathrm{h.c.}\right) \;+\; m_f\sum_{n=0}^{N-1}(-1)^n\phi_n^{(f)\dagger}\phi_n^{(f)}\right] + \frac{g^2}{2}\sum_{s=1}^{L}\left(-s+\frac{s^2}{N}\right)(1-\frac{1}{2}\delta_{s,L})\left(\sum_{n=0}^{N-1}\sum_{a=1}^{8}Q_n^{(a)}Q_{n+s}^{(a)}\right) . \tag{5}$$

Similar Hamiltonians that have used Gauss's law to remove gauge degrees of freedom in 1+1D lattice gauge theories with PBCs were used in Refs. [45, 51–55].

A. Truncating the Chromoelectric Interaction

In theories with a mass gap between the vacuum and first excited state, like 1+1D QCD, correlations in the vacuum decay exponentially between distant charges,

$$\sum_{a=1}^{8}\langle Q_n^{(a)}Q_{n+s}^{(a)}\rangle \;\sim\; e^{-c_1 s\, m_{\mathrm{hadron}}} , \tag{6}$$

where c_1 is a geometrical factor, and m_{hadron} is the mass of the lightest excitation in the vacuum sector, the scalar meson. Vacuum expectation values are denoted by $\langle\,.\,\rangle$. In previous work [56], the exponential decay of correlations between electric charges in the Schwinger model (one-dimensional $U(1)$ lattice gauge theory) was used to form an approximate interaction where charges interacting beyond a certain range were truncated.[4] A similar strategy is used here to truncate interactions between color charges separated by more than λ staggered sites as,

$$\hat{H}_{el}(\lambda) \;=\; \frac{g^2}{2}\sum_{s=1}^{\lambda}\left(-s+\frac{s^2}{2L}\right)(1-\frac{1}{2}\delta_{s,L})\left(\sum_{n=0}^{2L-1}\sum_{a=1}^{8}Q_n^{(a)}Q_{n+s}^{(a)}\right) . \tag{7}$$

This truncated interaction reduces the two-qubit gate count required for time evolution from scaling as $\mathcal{O}(N^2)$ to $\mathcal{O}(\lambda N)$. The quantum simulations performed on IonQ Forte-generation QPUs in Results used $\lambda = 1$.

B. The Weak Interaction

The weak interactions giving rise to single-β decay are modeled through a local vector-like four-Fermi operator [44],

$$\begin{aligned}
\hat{H}_{\beta}^{1+1} &= \frac{G}{\sqrt{2}}\int d^2x\,(\overline{\psi}_u\gamma^\mu\psi_d\;\overline{\psi}_e\gamma_\mu\mathcal{C}\psi_\nu + \mathrm{h.c.})\\
&\to \frac{G}{\sqrt{2}}\sum_{n\ \mathrm{even}}\Big[\left(\phi_n^{(u)\dagger}\phi_n^{(d)} + \phi_{n+1}^{(u)\dagger}\phi_{n+1}^{(d)}\right)\left(\chi_n^{(e)\dagger}\chi_{n+1}^{(\nu)} - \chi_{n+1}^{(e)\dagger}\chi_n^{(\nu)}\right)\\
&\qquad + \left(\phi_n^{(u)\dagger}\phi_{n+1}^{(d)} + \phi_{n+1}^{(u)\dagger}\phi_n^{(d)}\right)\left(\chi_n^{(e)\dagger}\chi_n^{(\nu)} - \chi_{n+1}^{(e)\dagger}\chi_{n+1}^{(\nu)}\right) + \mathrm{h.c.}\Big]\\
&\approx \frac{G}{\sqrt{2}}\sum_{n\ \mathrm{even}}\left(\phi_n^{(u)\dagger}\phi_n^{(d)}\phi_n^{(e)\dagger}\phi_{n+1}^{(\nu)} + \mathrm{h.c.}\right) ,
\end{aligned} \tag{8}$$

where $\mathcal{C}$ is the charge-conjugation operator, γ^μ are the gamma-matrices and G is the weak coupling constant (Fermi's constant). The first line shows the 1+1D interaction related to the low-energy charged-current weak interaction of the

[4] In $U(1)$ gauge theory, due to staggering, it is only the connected part of this correlation that falls exponentially. In $SU(3)$ gauge theory, the disconnected part of the correlation vanishes when evaluated in physical color singlet states.

Standard Model. The mapping of the fermion fields to a staggered lattice is shown in the second line. This necessarily includes contribution from both particles and anti-particles due to operator contractions. The results displayed in Fig. 2 in the main text are obtained using this interaction. The third line employs a "valence-fermion" approximation that only keeps the terms acting on the valence-quarks (no operators acting on anti-quark sites) and valence-leptons (no operators acting on neutrino or anti-electron sites). The results displayed in Fig. 3 in the main text are obtained using this approximation.

C. The Complete Spin Hamiltonian

The quantum simulations described in Results used $L = 2$ spatial sites and the truncated chromoelectric interaction in Supplementary Eq. (7) with $\lambda = 1$. The ordering of the fermionic degrees of freedom is shown in Fig. 4 in the main text. The JW transformation is used to map fermionic to spin operators that acts on qubits,

$$\phi_i^\dagger = \prod_{j<i} \left(-\hat{Z}_j\right) \hat{\sigma}_i^+ \quad , \quad \phi_i \; = \prod_{j<i} \left(-\hat{Z}_j\right) \hat{\sigma}_i^- \; , \tag{9}$$

where $\phi^\dagger$ and ϕ are fermionic creation and annihilation operators, $\hat{Z}$ is the Pauli-Z operator, and $\hat{\sigma}^+$ and $\hat{\sigma}^-$ are the spin-raising and spin-lowering operators.

After the JW transformation is applied, the complete spin Hamiltonian is

$$\begin{aligned}\hat{H}_{\text{quarks}} \to\; & \frac{1}{2}\sum_{n=0}^{3}\sum_{f=0}^{1}\sum_{c=0}^{2} m_f \left[(-1)^n \hat{Z}_{6n+3f+c} + \hat{I}\right] \\ & - \frac{1}{2}\sum_{n=0}^{2}\sum_{f=0}^{1}\sum_{c=0}^{2}\left[\hat{\sigma}^+_{6n+3f+c}\hat{Z}^5\hat{\sigma}^-_{6n+6+3f+c} + \text{h.c.}\right] + \frac{1}{2}\sum_{f=0}^{1}\sum_{c=0}^{2}\left[\hat{\sigma}^+_{18+3f+c}\hat{Z}^5\hat{\sigma}^-_{3f+c} + \text{h.c.}\right] \; ,\end{aligned} \tag{10a}$$

$$\begin{aligned}\hat{H}_{\text{leptons}} \to\; & \frac{1}{2}\sum_{n=0}^{3}\sum_{f=0}^{1} m_f \left[(-1)^n \hat{Z}_{24+2n+f} + \hat{I}\right] + \frac{1}{2}\sum_{l=0}^{1} m_M \left[\hat{\sigma}^+_{24+4l}\;\hat{Z}\hat{\sigma}^+_{26+4l} \;+\; \text{h.c.}\right] \\ & - \frac{1}{2}\sum_{n=0}^{2}\sum_{f=0}^{1}\left[\hat{\sigma}^+_{24+2n+f}\hat{Z}\hat{\sigma}^-_{26+2n+f} + \text{h.c.}\right] + \frac{1}{2}\sum_{f=0}^{1}\left[\hat{\sigma}^+_{30+f}\hat{Z}\hat{\sigma}^-_{24+f} + \text{h.c.}\right] \; ,\end{aligned} \tag{10b}$$

$$\hat{H}_{\text{glue}} \to -\frac{g^2}{2}\sum_{n=0}^{3}\frac{3}{4}\hat{Q}^{(a)}_n\hat{Q}^{(a)}_{n+1} \; , \tag{10c}$$

$$\begin{aligned}\hat{H}^{1+1}_\beta \to\; & \frac{G}{\sqrt{2}}\sum_{l=0}^{1}\sum_{c=0}^{2}\Big[\hat{\sigma}^-_{24+4l+2}\hat{\sigma}^+_{24+4l+1}\hat{\sigma}^-_{12l+3+c}\hat{Z}^2\hat{\sigma}^+_{12l+c} \;-\; \hat{\sigma}^+_{24+4l+3}\hat{Z}^2\hat{\sigma}^-_{24+4l}\hat{\sigma}^-_{12l+9+c}\hat{Z}^2\hat{\sigma}^+_{12l+6+c} \\ & - \hat{\sigma}^+_{24+4l+3}\hat{Z}^2\hat{\sigma}^-_{24+4l}\hat{\sigma}^-_{12l+3+c}\hat{Z}^2\hat{\sigma}^+_{12l+c} \;+\; \hat{\sigma}^-_{24+4l+2}\hat{\sigma}^+_{24+4l+1}\hat{\sigma}^-_{12l+9+c}\hat{Z}^2\hat{\sigma}^+_{12l+6+c} \\ & + \hat{\sigma}^+_{24+4l+1}\hat{\sigma}^-_{24+4l}\hat{\sigma}^+_{12l+6+c}\hat{Z}^2\hat{\sigma}^-_{12l+3+c} \;-\; \hat{\sigma}^+_{24+4l+3}\hat{\sigma}^-_{24+4l+2}\hat{\sigma}^-_{12l+9+c}\hat{Z}^8\hat{\sigma}^+_{12l+c} \\ & + \hat{\sigma}^+_{24+4l+1}\hat{\sigma}^-_{24+4l}\hat{\sigma}^-_{12l+9+c}\hat{Z}^8\hat{\sigma}^+_{12l+c} \;-\; \hat{\sigma}^+_{24+4l+3}\hat{\sigma}^-_{24+4l+2}\hat{\sigma}^+_{12l+6+c}\hat{Z}^2\hat{\sigma}^-_{12l+3+c} \;+\; \text{h.c.}\Big] \; .\end{aligned} \tag{10d}$$

Here, $\hat{H}_{\text{quarks}} + \hat{H}_{\text{leptons}} = \hat{H}_{\text{free}}$ (defined in Eq. 3 in the main text) and the hopping term between the last and first staggered site has a relative minus sign due to the JW mapping. The index l in $\hat{H}^{1+1}_\beta$ labels the spatial sites. We have used the short-hand notation $\hat{\sigma}_i\hat{Z}^{(j-i-1)}\hat{\sigma}_j = \hat{\sigma}_i(\prod_{k=i+1}^{j-1}\hat{Z}_k)\hat{\sigma}_j$. In $\hat{H}_{\text{quarks}}$, $m_0 = m_u$ and $m_1 = m_d$. In $\hat{H}_{\text{leptons}}$,

$m_0 = m_\nu$ and $m_1 = m_e$. The product of $SU(3)$ charges are

$$\sum_{a=1}^{8} \hat{Q}_n^{(a)}\, \hat{Q}_m^{(a)} = \sum_{f=0}^{1}\sum_{f'=0}^{1} \frac{1}{4}\Big[\, 2\Big(\hat{\sigma}^+_{6n+3f}\hat{\sigma}^-_{6n+3f+1}\hat{\sigma}^-_{6m+3f'}\hat{\sigma}^+_{6m+3f'+1} + \hat{\sigma}^+_{6n+3f}\hat{Z}_{6n+3f+1}\hat{\sigma}^-_{6n+3f+2}\hat{\sigma}^-_{6m+3f'}\hat{Z}_{6m+3f'+1}\hat{\sigma}^+_{6m+3f'+2} + \hat{\sigma}^+_{6n+3f+1}\hat{\sigma}^-_{6n+3f+2}\hat{\sigma}^-_{6m+3f'+1}\hat{\sigma}^+_{6m+3f'+2} + \text{h.c.}\Big) + \frac{1}{6}\sum_{c=0}^{2}\sum_{c'=0}^{2}(3\delta_{cc'}-1)\hat{Z}_{6n+3f+c}\hat{Z}_{6m+3f'+c'}\,\Big]\,. \tag{11}$$

For completeness, we provide the total electric charge $\hat{Q}_{\text{tot}}$ (quark + lepton) and the diagonal color charges (redness $\hat{r}$, blueness $\hat{b}$ and greenness $\hat{g}$),

$$\begin{aligned}
\hat{r} &= \frac{1}{2}\sum_{n=0}^{3}\sum_{f=0}^{1}\hat{Z}_{6n+3f}\ , \\
\hat{g} &= \frac{1}{2}\sum_{n=0}^{3}\sum_{f=0}^{1}\hat{Z}_{6n+1+3f}\ , \\
\hat{b} &= \frac{1}{2}\sum_{n=0}^{3}\sum_{f=0}^{1}\hat{Z}_{6n+2+3f}\ , \\
\hat{Q}_{\text{tot}} &= \frac{1}{2}\sum_{n=0}^{3}\sum_{f=0}^{1}\sum_{c=0}^{2} q_f\hat{Z}_{6n+3f+c} \;-\; \frac{1}{2}\sum_{n=0}^{3}\hat{Z}_{25+2n}\ ,
\end{aligned} \tag{12}$$

where $q_0 = 2/3$ is the electric charge of the up quark and $q_1 = -1/3$ is the electric charge of the down quark. The initial state $|\Delta^-\Delta^-\rangle$ states used in our quantum simulations has $r = g = b = 2$ and $Q_{\text{tot}} = -2$. These charges are conserved under time evolution, but can be violated by device errors. The results obtained from IonQ Forte Enterprise were post-selected to conserve these charges.

After the JW mapping, the complete charged-current weak interaction (modeled in 1+1D) is given in Supplementary Eq. (10d). It includes operators acting on both the valence- and sea-fermions, i.e., an up-quark operator can create an up-quark or it can annihilate an anti-up-quark, both actions change the up-quark number by one. For a complete simulation, all possible such actions should be included. However, retaining only the contributions from the operators acting on valence quarks is a well-defined approximation that can be implemented, reducing the required depth of quantum circuits. Supplementary Eq. (13) gives the Hamiltonian describing only the valence weak interactions, the approximation that we make in obtaining the results shown in Fig. 3 and shown in Table 2 in the main text,

$$\hat{H}^{1+1}_{\beta,\text{valence}} = \frac{G}{\sqrt{2}}\sum_{l=0}^{1}\sum_{c=0}^{2}\ \hat{\sigma}^-_{24+4l+2}\hat{\sigma}^+_{24+4l+1}\hat{\sigma}^-_{12l+3+c}\hat{Z}^2\hat{\sigma}^+_{12l+c}\quad . \tag{13}$$

Supplementary Note 4. Bootstrapping for Uncertainty Estimation

As the outputs of the quantum simulation we perform are computed with both post-selection and the non-linear filtering, estimating the uncertainty of the estimated outputs is non-trivial. We perform a bootstrap [57] estimation of these error bars based on the two sources of variation: the choice of circuit variants and the individual shots for each of those variants. For N_V variants and n_s shots per variant, we re-sample our measurements by taking the following steps:

1. Sample, with replacement, $N_V/2$ pairs of variants among the set of variant pairs used. (Recall from Methods C that the variants are generated in pairs, with each pair differing only by bit flips before measurement to symmetrize readout errors.)

2. Combine all pairs selected in the previous step into a set of N_V variants. For each of the resampled variants, sample (with replacement) n_s bit strings from the histograms of bit strings for each variant.

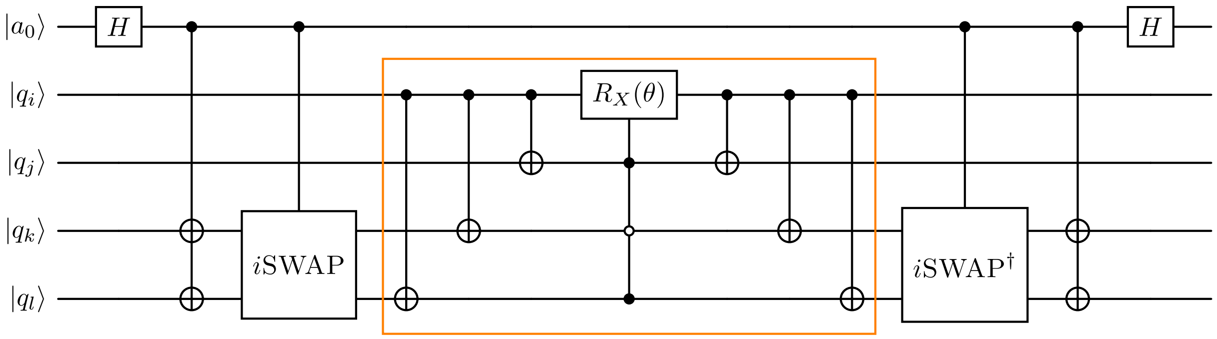

Supplementary Figure 3. Negated iSWAP-check placement around the unitary generated by the four-Fermi operator, $\theta\sigma_i^-\sigma_j^+\sigma_k^-\sigma_l^+$ + h.c. (the orange box), on $k-l$ qubits. Controlled iSWAP with two controlled NOTs commute with the four-Fermi operator and do not change the state of the ancilla $|a_0\rangle$ in the absence of errors.

3. Compute the simulation outputs for the resampled data, using the same post-selection and non-linear filtering described above.

By repeating this resampling procedure we construct a population of resampled outputs, and this population is used to estimate the standard-deviation of those outputs. For this work we used 10^5 bootstrap samples to estimate these standard-deviations. We then use these standard deviations as our reported error bars.

We note that this bootstrapping approach has a weakness: it does not account for the intentional structure of the choice of variants. Since the variants are chosen to have different biases due to noise in such a way that they should cancel, modeling them as random draws over-estimates the variance due to this selection. However, as modeling the precise uncertainty due to this bias cancellation would require an impractically detailed and accurate description of the device noise (including drift in that noise over time), we choose to use the random-draw model for bootstrapping and err on the side of over-estimating the uncertainties.

Supplementary Note 5. Error Mitigation with Flag Gadgets

Flag gadgets can be used to verify mid-circuit symmetries derived from the commutation properties of subcircuit blocks [58]. In quantum chemistry, as in QCD, one of the most common subcircuit blocks expresses particle-preserving unitaries, an extension of Givens rotations [59], which have multiple SWAP symmetries. For example, four-qubit Givens [60] rotations, which are equivalent to four-Fermi operators $\theta\sigma_i^-\sigma_j^+\sigma_k^-\sigma_l^+$ + h.c. up to a phase factor,[5] commute with SWAP gates applied on $i-k$ or $j-l$ qubit pairs and negated SWAP gates applied on $i-j$ or $k-l$ qubit pairs. Since four-Fermi operators also commute with R_{ZZ} gates, it means they also commute with iSWAP and negated iSWAP gates, as shown in Supplementary Fig. 3.

We chose iSWAP symmetry checks over R_{ZZ} and SWAP checks because of IonQ Forte and Forte Enterprise having low X- and Y-errors from crosstalk or under-rotation compared to phase errors. Optimal construction of symmetry checks such as iSWAP checks or negated iSWAP checks, which naively require 16 two-qubit gates, can be reduced to only 6 additional two-qubit gates per check (see Supplementary Fig. 4). We group together $\hat{H}_\beta^{1+1}$ terms applied on the same lepton qubits (light-brown circuit blocks in Supplementary Fig. 10) and apply negated iSWAP checks around them to ensure that the number of added gates (6 R_{ZZ} gates) is less than the number of gates in the checked subcircuit (33 R_{ZZ} gates). All iSWAP checks are applied on the lepton register. Used together with dynamical decoupling, mid-circuit symmetry checks improve the circuit fidelity upon post-selection, as shown in Supplementary Table 2.

Leakage and qubit loss errors in quantum computers lead to qubits becoming unresponsive to quantum gates. Undetected leakage errors can severely corrupt quantum computation, especially if they happen early in the circuit. In ion-trapped devices, leakage errors can happen due to the spontaneous emission and electronic transitions outside of the computational space. Some known solutions use custom-designed gates that target the extended subspace to

[5] Givens rotations (with real matrix elements) express unitary transformations by the exponential of $\theta\sigma_i^-\sigma_j^+\sigma_k^-\sigma_l^+$ − h.c. (note the minus sign).

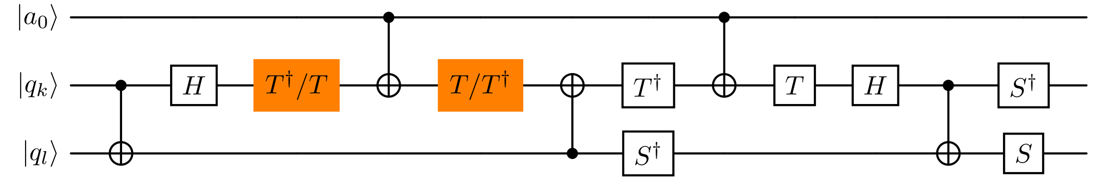

Supplementary Figure 4. Optimized construction of a controlled iSWAP (or a controlled negated iSWAP if the marked T gates are inverted) requires only five two-qubit gates. When placed on each side of the optimized Givens rotations, $k-l$ CNOTs cancel out leading to only 6 two-qubit gates overhead per check.

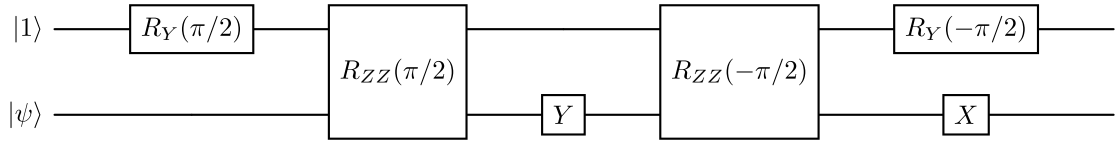

Supplementary Figure 5. The ancilla qubit (shown as the top wire) is a flag qubit that is prepared in state $|1\rangle$ (or $|0\rangle$). The target qubit on which the leakage is tested can be in any state $|\psi\rangle$ since the following sequence of gates does not affect it. The ancillary qubit ends up in state $|0\rangle$ (or $|1\rangle$) if the target qubit is in the computational space.

t	Error Mitigation	$\langle\hat{\mathcal{L}}\rangle_{m_M=0}$	$\langle\hat{\mathcal{L}}\rangle_{m_M=1.7}$	$\langle\hat{Q}_e\rangle_{m_M=0}$	$\langle\hat{Q}_e\rangle_{m_M=1.7}$
0.5	No EM	-0.04 ± 0.02	-0.05 ± 0.02	-0.12 ± 0.01	-0.10 ± 0.01
	DNL	-0.16 ± 0.02	-0.14 ± 0.02	-0.26 ± 0.02	-0.26 ± 0.02
	DNL + PS	-0.06 ± 0.08	-0.10 ± 0.07	-0.54 ± 0.04	-0.52 ± 0.05
	DNL + PS + FG	-0.06 ± 0.08	-0.09 ± 0.07	-0.52 ± 0.05	-0.54 ± 0.05
1.0	No EM	-0.04 ± 0.02	-0.01 ± 0.02	-0.17 ± 0.02	-0.16 ± 0.01
	DNL	-0.12 ± 0.03	-0.01 ± 0.02	-0.37 ± 0.02	-0.36 ± 0.02
	DNL + PS	0.04 ± 0.07	0.11 ± 0.08	-0.89 ± 0.06	-0.87 ± 0.06
	DNL + PS + FG	0.00 ± 0.06	0.12 ± 0.09	-0.90 ± 0.07	-0.86 ± 0.08
1.5	No EM	-0.02 ± 0.02	0.07 ± 0.01	-0.20 ± 0.02	-0.19 ± 0.01
	DNL	-0.10 ± 0.03	0.19 ± 0.02	-0.4 ± 0.02	-0.43 ± 0.02
	DNL + PS	0.09 ± 0.06	0.68 ± 0.11	-1.05 ± 0.06	-1.24 ± 0.06
	DNL + PS + FG	0.05 ± 0.05	0.59 ± 0.11	-1.04 ± 0.06	-1.25 ± 0.07
2.0	No EM	-0.02 ± 0.01	0.28 ± 0.01	-0.31 ± 0.01	-0.30 ± 0.01
	DNL	-0.03 ± 0.02	0.37 ± 0.02	-0.45 ± 0.01	-0.41 ± 0.01
	DNL + PS	0.13 ± 0.07	1.41 ± 0.09	-1.10 ± 0.05	-1.38 ± 0.05
	DNL + PS + FG	0.08 ± 0.07	1.43 ± 0.12	-1.13 ± 0.05	-1.41 ± 0.06

Supplementary Table 2. Forte experimental results of $\langle\hat{\mathcal{L}}\rangle$ and $\langle\hat{Q}_e\rangle$ for $m_M = 0$ and $m_M = 1.7$ at different evolution times t. The results are shown for four distinct levels of error mitigation (EM). The "No EM" data represents the raw experimental output. Applying debiasing with non-linear filtering yields the "DNL" results. The "DNL + PS" data further includes post-selection (PS) on the total charge Q_e without applying post-selection based on flag gadgets. Finally, the "DNL + PS + FG" data shows the fully mitigated results, which adds post-selection on flag gadgets (FG) and corresponds to the data in Table 2 in the main text.

which the leakage occurs [61]. We make use of a different approach with a combination of two fully entangling gates and single-qubit flips to flag leakage events when entangling fails [62, 63]. Our construction of this check is shown in Supplementary Fig. 5.

Supplementary Note 6. Additional Details on the Selection of Parameters

The following parameters are used in our simulations (in lattice units),

$$m_u = 1 \ , \ m_d = 1.5 \ , \ m_e = 0.1 \ , \ m_\nu = 1.5 \ , \ m_M = \{0.0, 1.7\} \ , \ g = 1 \ , \ G = 1 \ , \ L = 2 \ . \tag{14}$$

The corresponding spectra obtained from exact diagonalization using these parameters are given in Supplementary Table 3. The energies are calculated from the Hamiltonian without weak interactions, i.e., $\hat{H} = \hat{H}_{\text{free}} + \hat{H}_{\text{glue}} + \hat{H}_{\text{Maj}}$. The left columns in Supplementary Table 3 show that, for $m_M = 0$, the states after a β- and $\beta\beta$-decay are higher in energy than the initial state. The right columns show that, for $m_M = 1.7$, only the state after single β-decay is higher in energy; the state after $0\nu\beta\beta$ is lower in energy.[6] Therefore, these parameters engineer an energy landscape that is similar to that found in, for example, ^{76}Ge, i.e., states obtained via a single β-decay from $|\Delta^-\Delta^-\rangle$ are higher in energy. However, these are the energies in the absence of the β-decay interaction. Due to the size of the Fermi constant ($G = 1$), as well as finite-size effects, decays are still allowed for $m_M = 0$, even though they are energetically disfavored. Of course, with $m_M = 0$, lepton number is conserved, and neutrinoless decays cannot occur.

$m_M = 0$		$m_M = 1.7$	
State	Energy	State	Energy
$\|\Delta^-\Delta^-\rangle$	6.7332	$\|\Delta^-\Delta^-\rangle$	6.7332
$\|\Delta^-\Delta^0\rangle + e^- + \overline{\nu}$	7.7241	$\|\Delta^-\Delta^0\rangle + e^- + \overline{\nu}$	6.8742
$\|\Delta^0\Delta^0\rangle + 2e^- + 2\overline{\nu}$	9.9384	$\|\Delta^0\Delta^0\rangle + 2e^-$	6.6356

Supplementary Table 3. The energies of states (in lattice units) relevant to β-decays, $2\nu\beta\beta$-decays and $0\nu\beta\beta$-decays, with the parameters given in Supplementary Eq. (14).

Supplementary Note 7. Comments on the Road Ahead for Simulation Parameter Extrapolation(s)

The road map for quantum simulations of fundamental physics is anticipated to mirror that of classical lattice QCD calculations. Starting in the mid-1970's, lattice QCD simulations were performed using selection of truncations and unphysical parameters, including finite-volume, unphysical quark masses, finite lattice spacings, quenching (valence quark contributions only) followed by partial-quenching (valence and sea-quarks included, but with different Hamiltonians). As computational capabilities increased, lattice QCD simulations were increasingly able to be extrapolated to the physical point with finite-volume and continuum extrapolations. For a review of uncertainty quantification in lattice QCD calculations, see Ref. [64].

The current status of quantum simulations should be considered to be in the era of the 1970's lattice QCD simulations, but with the additional overhead of quantum computers producing observable dependent errors in simulations. Even after we evolve to the point of present day simulations, extrapolations in the parameters of the neutrino sector, from the simulation values to their physical (or limit values), will be required.[7] Given the extreme hierarchy of scales, such extrapolations are expected to be straightforward once simulations have been performed with sufficient fidelity.

A further added complication for the quantum simulations is the extraction of lifetimes from simulations. The time evolution of the change in final-state lepton number becomes exponential for large volumes and at late times. However, before these conditions are satisfied, the exponential decay is only approximate, from which the mean-lifetime must be extracted. This was studied in detail in 1+1D for β-decay in Ref. [44].

Supplementary Note 8. Debiasing with Non-linear Filtering

Debiasing with non-linear filtering (DNL) is a resource-efficient error-mitigation strategy that leverages circuit and device symmetries [73]. In this approach we prepare a number of variants of a quantum circuit that correspond to the

---

[6] The lepton vacuum for $m_M = 1.7$ and $m_M = 0$ are identical, with $\mathcal{L} = 0$. In general, for $m_M \neq 0$, $\mathcal{L}$ is not a good quantum number and, for example, the state labeled by $\overline{\nu}$ in the second row of the right side of Supplementary Table 3 has components with $\mathcal{L} = -1$ and $\mathcal{L} = +1$.

[7] Quantum simulations of neutrinos [65–72] are also being used to explore collective flavor dynamics that occur in high-density non-equilibrium environments, such as those formed in supernova.

same ideal computation but differ in the embedding of the algorithmic qubits to ions as well as gate decomposition and implementations. By choosing these variants to symmetrize the effects of the dominant hardware errors, the effect of those errors can be reduced in post processing by applying a filtering function.

This debiasing method can be highly efficient as these variants are carefully selected as opposed to being random draws (such as is done with standard twirling). For example, given a measurement bias between the $|0\rangle$ and $|1\rangle$ states in the hardware, one could always prepare an even number of variants grouped into pairs that are identical up to attaching NOT gates at the end of the circuit to the corresponding qubits. Undoing those NOT operations in postprocessing will then cancel out the measurement bias for each pair, assuming there is no significant drift in that bias while the pair of variants are collected. Another relevant symmetry available on the Forte-generation devices is the ability to freely remap algorithmic qubits to ions due to the all-to-all connectivity of the devices. This hardware feature makes it simple to symmetrize the different errors associated with the locations of each ion.

The algorithm for aggregation by non-linear filtering (outlined in Supplementary Fig. 6) is given below:

1. For each bit string obtain a sorted distribution of observed frequencies of this bit string by each twirled variant (Supplementary Fig. 6a).

2. Transpose the distribution of observed frequencies per variant to obtain the number of variants observing the given bit string with at least a specified frequency (Supplementary Fig. 6b). The normalized area under that curve corresponds to the frequency of observing a given bit string if the data from the variants are directly merged without filtering. Note that the variants are considered individually regardless of how they were generated.

3. Apply a threshold-based filtering function that discards bit strings not observed by the minimum number of variants. Higher thresholds allow for better mitigation of the biases introduced by the different impact of hardware noise across the variants, but are limited by the number of variants and shots taken (see Supplementary Note 9). Since the observables are calculated on the lepton register, the quark register was ignored at this step.

4. Calculate the aggregated probability estimates as the normalized area under the curve after this filtering has been applied.

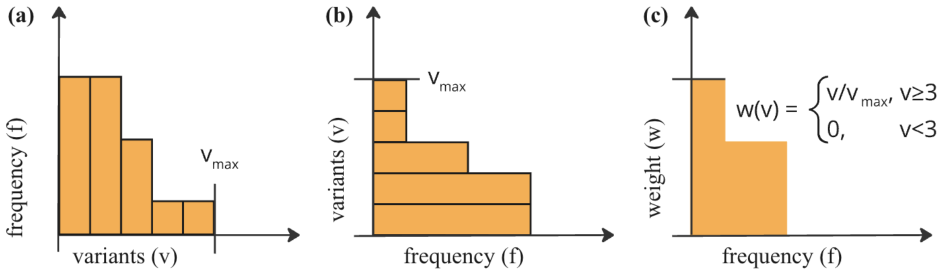

Supplementary Figure 6. Aggregation with non-linear filtering. For each measured bit string, a distribution of frequencies per variant (a) is sorted from largest to smallest to be converted into a function of the number of individual variants simultaneously observing the given bit string at a given frequency (b). Normalized area under that curve leads to the aggregated results that correspond exactly to the aggregation by simple averaging if no filter is applied. (c) Filtering out any contributions observed by fewer than a certain minimum number of variants (3 on the picture) rules out accidental and biased counts.

This algorithm reduces the effect of bit strings that appear more frequently in only a few variants. This is an effective error mitigation strategy as all of the variants correspond to the same ideal quantum circuit, meaning the appearance of bit strings with higher frequencies in only a small subset of the variant histograms is likely due to noise. The threshold parameter introduced in Step 3 determines the size of this subset. In other words, the computed probability estimation depends more strongly on how common a bit string is among the variants than its frequency in a given histogram, especially when working with small numbers of shots.

Since the post-selection of bit strings in this method relies on the overlap between different variants, it follows that in the worst-case scenario, finding a non-zero overlap would require exponentially many measurements. In the middle-case scenario, this effect can be mitigated by leveraging the properties of observable instead of trying to refine the original measurement statistics. For example, filtering can also be applied to binned subsets of bit strings to enhance the resolution of local observable if the shot count is low. The qubits not used for estimating that observable can

be ignored by binning the outputs based on the measurements of the relevant qubits. This approach was used when working with the high depth circuit discussed in Results. More symmetries can be used in larger scale simulations to increase the overlap between the variants. Binning can also be adjusted based on the targeted noise channels while others can be addressed with error detection.

Supplementary Note 9. Monte Carlo Method for Determining the Variant Count

When preparing to run the quantum simulation with circuit variants, we first need to determine how many variants to use and how many shots to take for each variant. A typical constraint on this optimization problem is the allocated runtime for the simulation on the quantum computer, and this is the constraint we used in making decisions for this project. Given a time budget, we estimate the number of shots that could be taken within that budget for a given number of variants based on the quantum gate times and related overheads. With this relationship between variants and shot count established, we then seek to minimize the expected absolute error in some output of a given combination. In this work we used the lepton number observable as the target output to optimize for.

Under the assumption that we do not know the ideal distribution of measured bit strings or the precise impact of hardware errors, we can approach this error estimate by marginalizing over both the ideal distribution and the distortion due to errors. This marginalization was performed with a Monte Carlo integration over prior probability distributions that describe this ignorance. We modeled these priors with symmetric Dirichlet distributions. That is, the probability vectors describing the output distributions were drawn from the probability density $f(\vec{p})$

$$f(\vec{p}) = \frac{\Gamma(d\,\overline{\alpha})}{\Gamma(\overline{\alpha})^d} \prod_{i=1}^{d} p_i^{\overline{\alpha}-1} \tag{15}$$

where d is the dimension of the probability vector $\vec{p}$ and $\overline{\alpha}$ is the concentration parameter. (Note that d can be much smaller than the full state-space of the quantum computer. An upper bound on the output dimension, for example, can be the number of shots taken.) To denote ignorance of the ideal probabilities we set $\overline{\alpha} = 1$ so that this density describes a uniform distribution over all possible probability vectors.

We used a simplified model that is assumed to be close to global depolarizing noise to model the impact of noise. For global depolarizing noise, when an error occurs the measurement is of the maximum entropy state, meaning that the probability distribution over bit strings is uniform. Given that we expect to find notable biases in the output due to noise, we do not want to enforce global depolarizing noise exactly, so we instead again used the symmetric Dirichlet distribution and set $\overline{\alpha} = 10$, which has the uniform probability vector as it's mode and but models error distributions that add some bias to global depolarizing noise.

With these distributions and device error rates we can sample possible (mock) sets of histograms for a set of circuit variants. Each such set is a single Monte Carlo sample, and is drawn with the following steps:

1. Draw a single mock "ideal" probability vector as well as a set of "noise" probability vectors.

2. Draw from a binomial distribution (with the probability set by the device's error rate) how many shots are noisy for each variant.

3. Construct a histogram for each variant by sampling the ideal distribution and variant-specific noise distribution a number of times determined in the previous step.

For each Monte Carlo sample the absolute error (after the error mitigation was applied) is then recorded. These sample absolute errors are then averaged over, resulting in approximate Monte Carlo integration to compute the expected absolute error in an observable. Note that this expected absolute error models the influence of both shot noise and hardware errors.

By focusing on the lepton number observable and evaluating this expected absolute error for a wide set of possible numbers of variants, we settled on using 64 variants with 150 shots each for circuits run on Forte Enterprise. This choice was not made solely based on the expected absolute error calculation but also incorporated previous experiments with the DNL error mitigation method to select among the possible variant counts that were predicted to have a good performance.

We note that the the actual number of variants used was higher (see Results) as we ended up being able to allocate additional system time for this project beyond the original estimate.

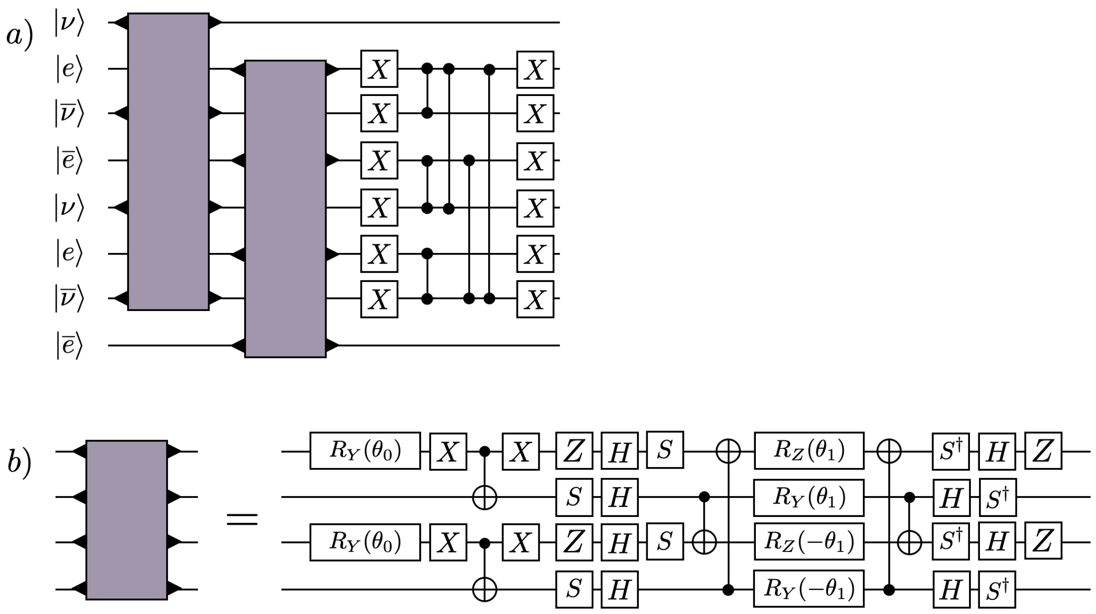

Supplementary Figure 7. The circuits for preparing the two-flavor lepton vacuum on $L = 2$. The circuit in a) prepares the two-flavor vacuum using the one-flavor vacuum preparation circuit block defined in b). The parameters $\{\theta_0, \theta_1\}$ are determined by variationally minimizing the one-flavor lepton Hamiltonian, and are given in Supplementary Eq. (16) for $m_e = 0.1$ and $m_\nu = 1.5$. The barbells denote CZ gates and the ►-symbols mark which qubits the circuit blocks are acting on.

Supplementary Note 10. Quantum Circuits for State Preparation and Time Evolution

In this supplementary note, we present the techniques used to construct the quantum circuits used in this work. This includes circuits to prepare $|\psi_{\text{init}}\rangle = |\psi_{\text{vac}}^{(\text{lep})}\rangle|\Delta^-\Delta^-\rangle$, and implement Trotterized time evolution. The circuits were further optimized when transpiling to IonQ's native gate set using the IonQ circuit compiler.

A. State Preparation

The initial two-baryon state is $|\psi_{\text{init}}\rangle = |\psi_{\text{vac}}^{(\text{lep})}\rangle|\Delta^-\Delta^-\rangle$, where $|\psi_{\text{vac}}^{(\text{lep})}\rangle$ is the ground state of $\hat{H}_{\text{leptons}}$ given in Supplementary Eq. (10b). Circuits for preparing the lepton vacuum on $L = 2$ spatial sites with PBCs are shown in Supplementary Fig. 7. This circuit uses layers of Givens rotations followed by fermionic SWAPs to exactly prepare the free fermion ground state [74–76]. The construction of this circuit follows several principles. First, the relative fermionic phases between electrons and neutrinos are pushed to the end of the circuit. This allows the circuits that initialize the electron and neutrino vacuums to be determined separately, since $|\psi_{\text{vac}}^{(\text{lep})}\rangle = |\psi_{\text{vac}}^{(e)}\rangle|\psi_{\text{vac}}^{(\nu)}\rangle$ if the fermionic statistics are ignored. The electron and neutrino ground states are real, translationally invariant and have equal fermion and anti-fermion occupation. The circuit in Supplementary Fig. 7b) is able to reach all such states, and it does so with two components. The first component applies an R_Y rotation on the first qubit of each spatial lattice site followed by $\hat{X}$ on the second qubit controlled on the first qubit being in state $|0\rangle$. This ensures that the initial state is real and has equal fermion and anti-fermion occupations. The second component produces all possible lepton-number conserving, real two-qubit transformations that the first layer does not account for. This is done using a pair of $(\hat{X}\hat{Y} - \hat{Y}\hat{X})$ rotations. The $(\hat{X}\hat{Y} - \hat{Y}\hat{X})$ rotation that wraps around the lattice has a relative minus sign due to the minus sign in the JW kinetic term. The resulting circuit exactly prepares the ground state and is parameterized by two angles $\{\theta_0, \theta_1\}$ that depend on the value of the fermion mass. For the masses chosen in Supplementary Eq. (14), the variational parameters are determined by minimizing the energy of the one-flavor lepton Hamiltonian, and are

$$\{\theta_0, \theta_1\}_{m_e=0.1} = \{0.7356, -1.2030\} \qquad \{\theta_0, \theta_1\}_{m_\nu=1.5} = \{0.2940, -1.4238\} \ . \tag{16}$$

Note that the parameters do not depend on the Majorana mass because $|\psi_{\text{vac}}^{(\nu)}\rangle$ is the same for the $m_\nu = 1.5$, $m_M = 0$ and $m_\nu = 1.5$, $m_M = 1.7$ used in this work. The fermionic statistics are included at the end of the circuit using the CZ network in Supplementary Fig. 7a).

The initial state in the QCD sector is $|\Delta^-\Delta^-\rangle$, which, as mentioned in Methods A, factorizes between the up- and down-quark sectors,[8]

$$|\Delta^-\Delta^-\rangle \;=\; |d_r d_g d_b\, d_r d_g d_b\rangle |\psi^{(u)}_{\text{vac}}\rangle \ . \tag{17}$$

The $|d_r d_g d_b\, d_r d_g d_b\rangle$ state is trivial to prepare, and circuits for approximately preparing $|\psi^{(u)}_{\text{vac}}\rangle$ can be determined with the SC-ADAPT-VQE algorithm [56, 77]. SC-ADAPT-VQE determines low-depth scalable circuits that efficiently prepare states with localized correlations using symmetries and hierarchies in length scales to define a variational circuit ansatz. These circuits are optimized to minimize the energy using ADAPT-VQE [78] running on a classical computer. Here, SC-ADAPT-VQE is used to determine circuits that prepare $|\psi^{(u)}_{\text{vac}}\rangle$. The QCD Hamiltonian describing a single quark flavor is similar to Supplementary Eq. (5),

$$\begin{aligned}\hat{H} = \sum_{c=0}^{2}\Bigg\{\frac{1}{2}\sum_{n=0}^{2L-1} m_u\left[(-1)^n\hat{Z}_{3n+c}+\hat{I}\right] \;+\; \frac{1}{2}\sum_{n=0}^{2L-2}\left[\hat{\sigma}^+_{3n+c}\hat{Z}^2\hat{\sigma}^-_{3n+3+c}+\text{h.c.}\right]+\frac{1}{2}(-1)^{L+1}\left[\hat{\sigma}^+_{6L-3+c}\hat{Z}^2\hat{\sigma}^-_{c}+\text{h.c.}\right]\Bigg\}\\ -\frac{g^2}{2}\sum_{n=0}^{2L-1}\sum_{a=1}^{8}\sum_{s=1}^{L}\left(s-\frac{s^2}{2L}\right)\left(1-\frac{\delta_{s,L}}{2}\right)\hat{Q}^{(a)}_n\hat{Q}^{(a)}_{n+s} \ ,\end{aligned} \tag{18}$$

where

$$\begin{aligned}\sum_{a=1}^{8}\hat{Q}^{(a)}_n\,\hat{Q}^{(a)}_m = \frac{1}{4}\Bigg[\;2\big(\hat{\sigma}^+_{3n}\hat{\sigma}^-_{3n+1}\hat{\sigma}^-_{3m}\hat{\sigma}^+_{3m+1}+\hat{\sigma}^+_{3n}\hat{Z}_{3n+1}\hat{\sigma}^-_{3n+2}\hat{\sigma}^-_{3m}\hat{Z}_{3m+1}\hat{\sigma}^+_{3m+2}+\hat{\sigma}^+_{3n+1}\hat{\sigma}^-_{3n+2}\hat{\sigma}^-_{3m+1}\hat{\sigma}^+_{3m+2}+\text{h.c.}\big)\\ +\frac{1}{6}\sum_{c=0}^{2}\sum_{c'=0}^{2}(3\delta_{cc'}-1)\hat{Z}_{3n+c}\hat{Z}_{3m+c'}\Bigg] \ .\end{aligned} \tag{19}$$

In ADAPT-VQE, the unitaries that prepare the vacuum are constructed from a pool of Hermitian operators $\{\hat{O}\}$. For vacuum preparation, the operators in the pool respect translational invariance, global $SU(3)$, baryon number, parity, charge conjugation and time reversal. The easiest way to construct operators that conserve these symmetries is by using terms in the Hamiltonian. However, terms in the Hamiltonian are real and their unitary evolution $e^{i\theta\hat{O}}$ is not optimal for preparing the vacuum, which is a real wavefunction. A good starting point is the commutator of terms in the Hamiltonian (multiplied by "i" to make them hermitian).[9] This can be generalized to $SU(3)$, and a suitable pool of operators is

$$\begin{aligned}\{\hat{O}\} \;&=\; \{\hat{O}_{mh}(s)\} \ ,\\ \hat{O}_{mh}(s) &= i\left[\hat{\Theta}_m,\hat{\Theta}_h(s)\right] \ ,\\ \hat{\Theta}_m &= \frac{1}{2}\sum_{n=0}^{N-1}\sum_{c=0}^{2}(-1)^n\hat{Z}_{3n+c} \ ,\\ \hat{\Theta}_h(s) &= \sum_{n=0}^{N-1}\sum_{c=0}^{2}v(n,s)(1-\frac{1}{2}\delta_{s,L})\left[\hat{\sigma}^+_{3n+c}\hat{Z}^{3s-1}\hat{\sigma}^-_{3(n+s)+c}+\text{h.c.}\right] \ ,\end{aligned} \tag{20}$$

where $\hat{\Theta}_h(s)$ is an operator that hops s staggered sites. The phase $v(n,s)=(\pm 1)^{L+1}$ with $(+)$ if $3(n+s)<3N-1$ and $(-)$ if $3(n+s)>3N-1$ comes from the minus sign in the JW mapped kinetic term. The range of s is $s\in\{1,3,\ldots,L\}$, and only s odd operators are generated as a consequence of charge conjugation symmetry. This pool of operators creates mesonic (quark-antiquark) excitations, and are found to provide rapid convergence to the vacuum for the parameter regime considered. The initial state for the SC-ADAPT-VQE preparation of the vacuum is the strong-coupling vacuum $|\Omega_0\rangle = |000\,111\rangle^{\otimes L}$. Surprisingly, as it is not the case with OBCs, the $\hat{O}_{mh}(s)$ with different (odd) s commute with each other, i.e., $[\hat{O}_{mh}(s),\hat{O}_{mh}(s')]=0$. Therefore, in the absence of Trotter errors, SC-ADAPT-VQE

---

[8] There are extra minus signs in the u-quark vacuum due to u-quarks hopping over three d-quarks.

[9] For preparing the vacuum of the Schwinger model, an operator pool generated from the algebra of the non-interacting Hamiltonian was found to be effective [56, 77, 79].

L \ step	δE 0	1	2	3	$\mathcal{I}_L$ 0	1	2	3
2	0.3462	0.0002			0.1492	0.00004		
3	0.3498	0.0014	0.0002		0.1431	0.0006	0.0001	
4	0.3471	0.0007	0.0002		0.1293	0.0003	0.0001	
5	0.3459	0.0007	0.00023	0.00022	0.1187	0.0003	0.000061	0.000064

Supplementary Table 4. The convergence of up to three steps of the SC-ADAPT-VQE algorithm for $L = 2 - 5$ and $m = g = 1$. For $L = 2$, SC-ADAPT-VQE has converged after one step, for $L = 3$ and $L = 4$, SC-ADAPT-VQE has converged after two steps, and for $L = 5$ it has converged after three steps. The deviation in the energy δE and the infidelity density $\mathcal{I}_L$ are defined in the text. Step zero represents the initial state $|\psi_{\text{ans}}\rangle = |\Omega_0\rangle$.

L \ θ_i	$\hat{O}_{mh}(1)$	$\hat{O}_{mh}(3)$	$\hat{O}_{mh}(5)$
2	0.1705		
3	0.1755	−0.0137	
4	0.1732	−0.0058	
5	0.1723	−0.0059	0.0008

Supplementary Table 5. The order of the operators $\hat{O}$ and variational parameters θ_i used to prepare the SC-ADAPT-VQE vacuum in 1+1D QCD with one flavor of quark. Shown are results for $L = 2 - 5$ and $m = g = 1$.

converges once all values of s are explored ($\lceil L/2 \rceil$ steps). Trotter errors will break this and SC-ADAPT-VQE may require more steps to converge.

Two quantities are used to quantify the quality of the SC-ADAPT-VQE prepared vacuum $|\psi_{\text{ans}}\rangle$. The first is the deviation in the energy of the ansatz state E_{ans} compared to the true vacuum energy E_{vac},

$$\delta E = \frac{E_{\text{vac}} - E_{\text{ans}}}{E_{\text{vac}}} \ , \tag{21}$$

and the second is the infidelity density,

$$\mathcal{I}_L = \frac{1}{L}\left(1 - |\langle \psi_{\text{vac}} | \psi_{\text{ans}} \rangle|^2\right) \ . \tag{22}$$

Results for the energy and infidelity density for $m = g = 1$ (with a correlation length of $\xi \propto m_{\text{hadron}}^{-1} = 0.34$ staggered sites, where m_{hadron} is the lightest hadron mass) are given in Supplementary Table 4. The sequence of operators and their corresponding variational parameters are given in Supplementary Table 5. As expected, SC-ADAPT-VQE initially builds out short-range correlations (small s), which have the largest variational parameters. The variational parameters are converging with the expected $e^{-L/\xi}$ scaling and could be robustly extrapolated to prepare the vacuum for large L. As mentioned above, the SC-ADAPT-VQE algorithm with this pool would converge after $\lceil L/2 \rceil$ steps in the absence of Trotterization errors. We find that the Trotter errors are small, and our implementation of SC-ADAPT-VQE also converges after $\lceil L/2 \rceil$ steps using a convergence criteria of $|\langle \psi_{\text{ans}} | [\hat{H}, \hat{O}] | \psi_{\text{ans}} \rangle| \leq 10^{-5}$. Despite rapid convergence, the ultimate wavefunction fidelity exceeds that required for near-term quantum simulations.

The circuits that implement the unitary evolution of the operators in Supplementary Eq. (20) are similar to those used for preparing the Schwinger model vacuum in Ref. [77]. Those circuits utilized the "X"-circuit design of Ref. [80] to minimize circuit depth on a device with nearest-neighbor connectivity. However, for devices with all-to-all connectivity like IonQ Forte, the technique using CZs that is described in Supplementary Note 10 B below is superior. An example of the circuit that prepares the SC-ADAPT-VQE vacuum for one quark flavor with $L = 2$ is shown in Supplementary Fig. 8a). This circuit has a CNOT depth of 8 (compared to 11 with nearest-neighbor connectivity).

The circuits that prepare $|\psi_{\text{vac}}^{(u)}\rangle$ on $L = 2$ can immediately be used to prepare $|\Delta^-\Delta^-\rangle$. The down quarks sites are fully occupied, which causes the signs of the variational parameters in the $|\psi_{\text{vac}}^{(u)}\rangle$ preparation circuit to be negated.

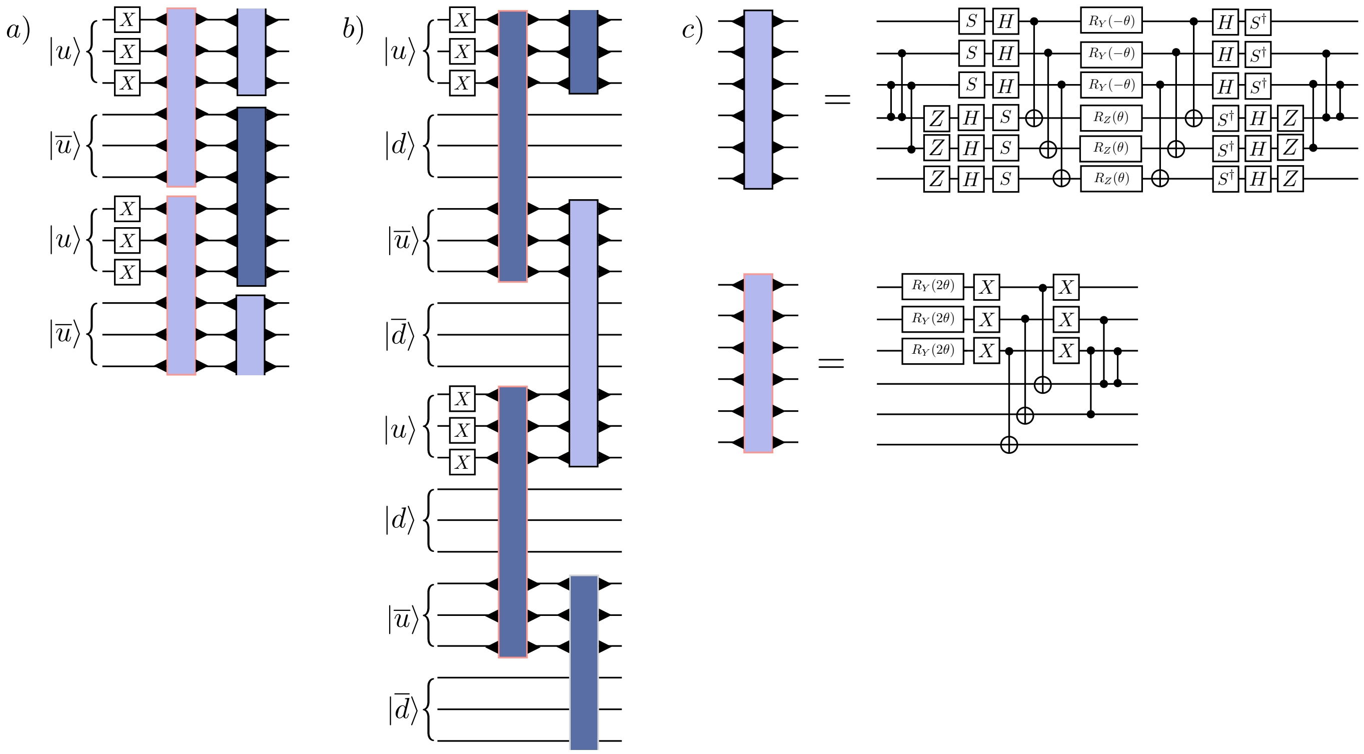

Supplementary Figure 8. The SC-ADAPT-VQE circuits used for state preparation in $1+1$D QCD on a $L=2$ lattice. The circuit in a) prepares $|\psi_{\text{vac}}^{(u)}\rangle$, and the circuit in b) prepares $|\Delta^-\Delta^-\rangle$. The circuits in c) implement $e^{-i\frac{\theta}{2}(\hat{X}\hat{Z}\hat{Z}\hat{Y}-\hat{Y}\hat{Z}\hat{Z}\hat{X})}$ and the pink-outlined circuit blocks are a simplification of the black outlined circuit blocks when acting on the strong-coupling vacuum initial state $|\Omega_0\rangle$. The dark purple blocks are the same as the light purple ones except with $\theta \to -\theta$. The value of θ for $m=g=1$ and $L=2$ is given in Supplementary Table 5. The barbells denote CZ gates and the ▶-symbols mark which qubits the circuit blocks are acting on.

The circuits used to prepare the $|\Delta^-\Delta^-\rangle$ state are shown in Supplementary Fig. 8b). The initial state for our quantum simulations $|\psi_{\text{init}}\rangle = |\psi_{\text{vac}}^{(u)}\rangle|\Delta^-\Delta^-\rangle$ is prepared by applying the circuit in Supplementary Fig. 8b) to qubits $q_0, q_1, \ldots, q_{23}$ and the lepton vacuum initialization circuit in Supplementary Fig. 7a) to qubits $q_{24}, q_{25}, \ldots, q_{31}$ (see layout in Fig. 4 in the main text).

B. Time Evolution

In our previous work, circuits for digitizing the time-evolution operator were developed, making use of an ancilla to store partial parities of the JW strings [44, 81]. An improved method for creating these circuits without an ancilla will be presented here [76, 82, 83]. These circuits are arranged to maximize parallelization, hence reducing the circuit depth. The idea is to design the circuits without the JW $\hat{Z}$ strings, and then add them in at the end. Important circuit identities are:

$$\begin{aligned} CZ(\hat{Y}\otimes\hat{I})CZ &= \hat{Y}\otimes\hat{Z} \ , \\ CZ(\hat{X}\otimes\hat{I})CZ &= \hat{X}\otimes\hat{Z} \ , \\ (CZ)^2 &= \hat{I} \ , \\ CZ_{i,i+1} &= CZ_{i+1,i} \ . \end{aligned} \tag{23}$$

Making use of these identities allows the JW $\hat{Z}$s to be put in by sandwiching the circuit with a sequence of CZs. An illustrative example of this method was given in Fig. 5 in the main text. The extension of this method to generate a circuit that implements the quark kinetic term in Supplementary Eq. (10a) is shown in Supplementary Fig. 9b), that implements the lepton kinetic term in Supplementary Eq. (10b) is shown in Supplementary Fig. 9c) and that

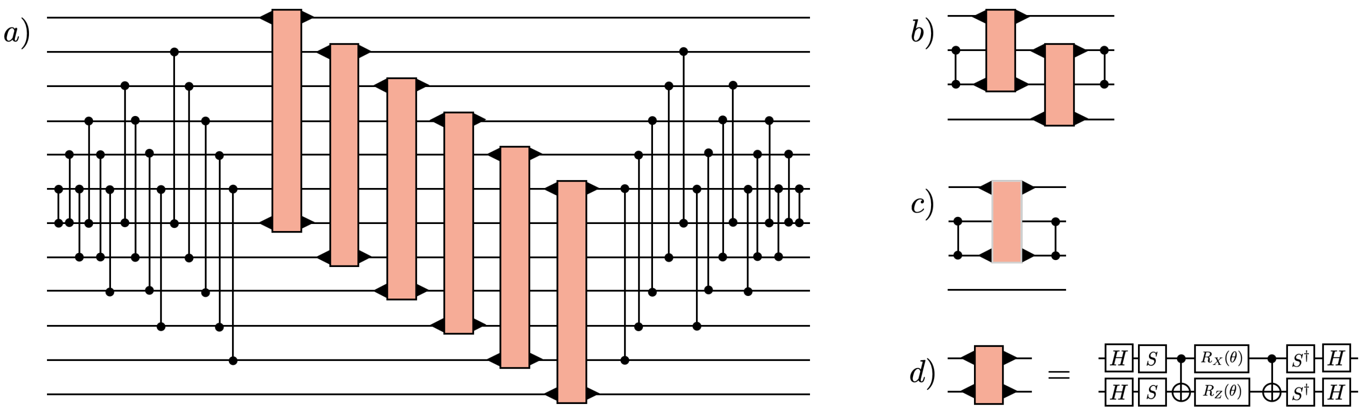

Supplementary Figure 9. Quantum circuits that implement the unitary evolution from the kinetic and Majorana mass terms in the Hamiltonian. The barbells denote CZ gates and the ►-symbols on the orange blocks mark the qubits that are acted on. a) A quantum circuit that implements the unitary evolution of the quark kinetic term in Supplementary Eq. (10a) across one spatial site. b) A quantum circuit that implements the unitary evolution of the lepton kinetic term in Supplementary Eq. (10b) across one spatial site. c) A quantum circuit that implements the unitary evolution of the neutrino Majorana mass term in Supplementary Eq. (10b) across one spatial site. The light-gray border on the circuit blocks denotes that the the rotation in d) is $R_X(-\theta)$. d) The definition of the light orange circuit block that implements $e^{-i\theta(\hat{X}\hat{X}+\hat{Y}\hat{Y})/2}$.

implements the Majorana mass term in Supplementary Eq. (10b) is shown in Supplementary Fig. 9d). Additionally, combining this method with the circuits from Refs. [44, 81, 84], allows the weak interactions in Supplementary Eq. (10d) to be implemented. This is shown in Supplementary Fig. 10 and significantly reduces the circuit depth relative to the circuits in Ref. [44]. For the strong-interaction terms in Supplementary Eq. (10c), we use the circuits from Ref. [81].

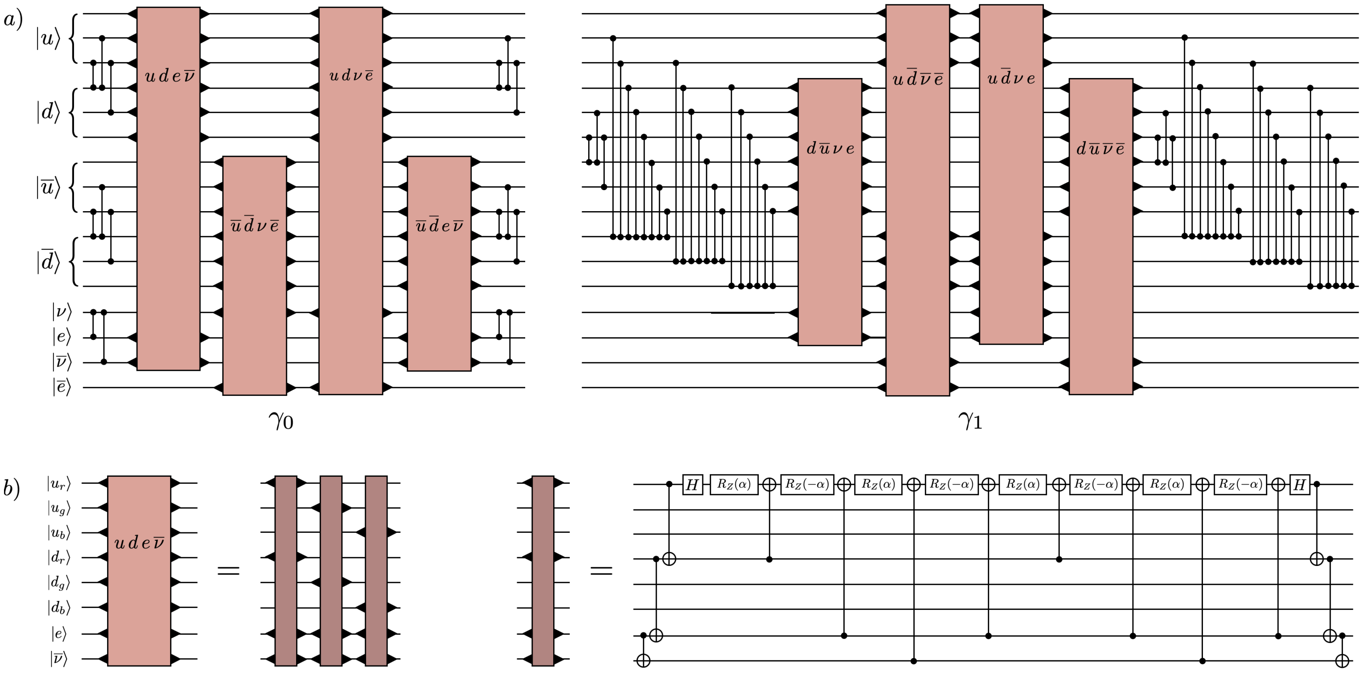

Supplementary Figure 10. a) The quantum circuits used to implement the unitary evolution from the β-decay interaction in Supplementary Eq. (10d) across one spatial site. The barbells denote CZ gates and the ▶-symbols on the light-brown box mark the qubits that are acted on. The circuits have been split into the γ_0 terms (first and second lines of H_β in Supplementary Eq. (10d)), and the γ_1 terms (third and fourth lines of H_β in Supplementary Eq. (10d)). The circuit blocks are labeled by the fermion sites that they act on and have been ordered to allow for maximum parallelizability. b) The definition of the light-brown circuit block using the GHZ diagonalization circuits (dark-brown circuit block) that implements $e^{-4i\alpha(\hat{\sigma}_i^+\hat{\sigma}_j^-\hat{\sigma}_k^+\hat{\sigma}_l^- + \text{h.c.})}$. This example is for the $ude\overline{\nu}$ term, and the rotation angle is $\alpha = \sqrt{2}Gt/8$. The other terms implement permutations of this circuit, that depend on the ordering of $\{i, j, k, l\}$ in $(\hat{\sigma}_i^+\hat{\sigma}_j^-\hat{\sigma}_k^+\hat{\sigma}_l^- + \text{h.c.})$.

[1] I. Esteban, M. C. Gonzalez-Garcia, M. Maltoni, I. Martinez-Soler, J. a. P. Pinheiro, and T. Schwetz, NuFit-6.0: updated global analysis of three-flavor neutrino oscillations, JHEP **12**, 216, arXiv:2410.05380 [hep-ph].

[2] E. Majorana, Teoria simmetrica dell'elettrone e del positrone, Nuovo Cim. **14**, 171 (1937).

[3] G. Racah, Sulla simmetria tra particelle e antiparticelle, Nuovo Cim. **14**, 322 (1937).

[4] Y. B. Zel'dovich and M. Y. Khlopov, Study of the neutrino mass in a double β decay, JETP **34**, 148 (1981).

[5] G. Anton *et al.* (EXO-200), Search for Neutrinoless Double-β Decay with the Complete EXO-200 Dataset, Phys. Rev. Lett. **123**, 161802 (2019), arXiv:1906.02723 [hep-ex].

[6] M. Agostini *et al.* (GERDA), Final Results of GERDA on the Search for Neutrinoless Double-β Decay, Phys. Rev. Lett. **125**, 252502 (2020), arXiv:2009.06079 [nucl-ex].

[7] D. Q. Adams *et al.* (CUORE), Search for Majorana neutrinos exploiting millikelvin cryogenics with CUORE, Nature **604**, 53 (2022), arXiv:2104.06906 [nucl-ex].

[8] S. Abe *et al.* (KamLAND-Zen), Search for the Majorana Nature of Neutrinos in the Inverted Mass Ordering Region with KamLAND-Zen, Phys. Rev. Lett. **130**, 051801 (2023), arXiv:2203.02139 [hep-ex].

[9] O. Azzolini *et al.* (CUPID), Final Result on the Neutrinoless Double Beta Decay of ^{82}Se with CUPID-0, Phys. Rev. Lett. **129**, 111801 (2022), arXiv:2206.05130 [hep-ex].

[10] I. J. Arnquist *et al.* (Majorana), Final Result of the Majorana Demonstrator's Search for Neutrinoless Double-β Decay in ^{76}Ge, Phys. Rev. Lett. **130**, 062501 (2023), arXiv:2207.07638 [nucl-ex].

[11] S. Abe *et al.* (KamLAND-Zen), Search for Majorana Neutrinos with the Complete KamLAND-Zen Dataset (2024), arXiv:2406.11438 [hep-ex].

[12] A. Agrawal *et al.* (AMoRE), Improved Limit on Neutrinoless Double Beta Decay of Mo100 from AMoRE-I, Phys. Rev. Lett. **134**, 082501 (2025), arXiv:2407.05618 [nucl-ex].

[13] H. Acharya *et al.* (LEGEND), First Results on the Search for Lepton Number Violating Neutrinoless Double Beta Decay with the LEGEND-200 Experiment (2025), arXiv:2505.10440 [hep-ex].

[14] C. Adams *et al.*, Neutrinoless Double Beta Decay (2022), arXiv:2212.11099 [nucl-ex].

[15] M. Agostini, G. Benato, J. A. Detwiler, J. Menéndez, and F. Vissani, Toward the discovery of matter creation with neutrinoless $\beta\beta$ decay, Rev. Mod. Phys. **95**, 025002 (2023), arXiv:2202.01787 [hep-ex].

[16] A. Barabash, Double beta decay experiments: Recent achievements and future prospects, Universe **9**, 290 (2023).

[17] NuPECC Long Range Plan 2024 for European Nuclear Physics (2025), arXiv:2503.15575 [nucl-ex].

[18] R. Mohapatra, Particle physics implications of neutrinoless double beta decay, Nucl. Phys. B Proc. Suppl. **77**, 376–385 (1999), arXiv:hep-ph/9808284.

[19] M. J. Savage, Pionic matrix elements in neutrinoless double Beta decay, Phys. Rev. C **59**, 2293 (1999), arXiv:nucl-th/9811087.

[20] V. Cirigliano *et al.*, Neutrinoless Double-Beta Decay: A Roadmap for Matching Theory to Experiment (2022), arXiv:2203.12169 [hep-ph].

[21] W. C. Haxton and G. J. Stephenson, Double beta Decay, Prog. Part. Nucl. Phys. **12**, 409 (1984).

[22] G. Prezeau, M. Ramsey-Musolf, and P. Vogel, Neutrinoless double beta decay and effective field theory, Phys. Rev. D **68**, 034016 (2003), arXiv:hep-ph/0303205.

[23] P. E. Shanahan, B. C. Tiburzi, M. L. Wagman, F. Winter, E. Chang, Z. Davoudi, W. Detmold, K. Orginos, and M. J. Savage, Isotensor Axial Polarizability and Lattice QCD Input for Nuclear Double-β Decay Phenomenology, Phys. Rev. Lett. **119**, 062003 (2017), arXiv:1701.03456 [hep-lat].

[24] B. C. Tiburzi, M. L. Wagman, F. Winter, E. Chang, Z. Davoudi, W. Detmold, K. Orginos, M. J. Savage, and P. E. Shanahan (NPLQCD Collaboration), Double-β decay matrix elements from lattice quantum chromodynamics, Phys. Rev. D **96**, 054505 (2017), arXiv:1702.02929 [hep-lat].

[25] V. Cirigliano, W. Dekens, J. de Vries, M. L. Graesser, E. Mereghetti, S. Pastore, and U. van Kolck, New leading contribution to neutrinoless double-β decay, Phys. Rev. Lett. **120**, 202001 (2018).

[26] V. Cirigliano, W. Dekens, J. De Vries, M. L. Graesser, E. Mereghetti, S. Pastore, M. Piarulli, U. Van Kolck, and R. B. Wiringa, Renormalized approach to neutrinoless double-β decay, Phys. Rev. C **100**, 055504 (2019), arXiv:1907.11254 [nucl-th].

[27] D. Castillo, L. Jokiniemi, P. Soriano, and J. Menéndez, Neutrinoless $\beta\beta$ decay nuclear matrix elements complete up to N2LO in heavy nuclei, Phys. Lett. B **860**, 139181 (2025), arXiv:2408.03373 [nucl-th].

[28] B. C. Tiburzi, M. L. Wagman, F. Winter, E. Chang, Z. Davoudi, W. Detmold, K. Orginos, M. J. Savage, and P. E. Shanahan, Double-β Decay Matrix Elements from Lattice Quantum Chromodynamics, Phys. Rev. D **96**, 054505 (2017), arXiv:1702.02929 [hep-lat].

[29] V. Cirigliano, W. Detmold, A. Nicholson, and P. Shanahan, Lattice QCD Inputs for nuclear double beta decay, Prog. Part. Nucl. Phys. **112**, 103771 (2020), arXiv:2003.08493 [nucl-th].

[30] A. Nicholson *et al.*, Heavy physics contributions to neutrinoless double beta decay from QCD, Phys. Rev. Lett. **121**, 172501 (2018), arXiv:1805.02634 [nucl-th].

[31] X.-Y. Tuo, X. Feng, and L.-C. Jin, Long-distance contributions to neutrinoless double beta decay $\pi^- \to \pi^+ ee$, Phys. Rev. D **100**, 094511 (2019), arXiv:1909.13525 [hep-lat].

[32] Z. Davoudi and S. V. Kadam, Two-neutrino double-β decay in pionless effective field theory from a Euclidean finite-volume correlation function, Phys. Rev. D **102**, 114521 (2020), arXiv:2007.15542 [hep-lat].

[33] Z. Davoudi and S. V. Kadam, Path from Lattice QCD to the Short-Distance Contribution to $0\nu\beta\beta$ Decay with a Light Majorana Neutrino, Phys. Rev. Lett. **126**, 152003 (2021), arXiv:2012.02083 [hep-lat].

[34] W. Detmold, W. I. Jay, D. J. Murphy, P. R. Oare, and P. E. Shanahan, Neutrinoless double beta decay from lattice QCD: The short-distance $\pi^- \to \pi^+ e^- e^-$ amplitude, Phys. Rev. D **107**, 094501 (2023), arXiv:2208.05322 [hep-lat].

[35] Z. Davoudi, W. Detmold, Z. Fu, A. V. Grebe, W. Jay, D. Murphy, P. Oare, P. E. Shanahan, and M. L. Wagman (NPLQCD), Long-distance nuclear matrix elements for neutrinoless double-beta decay from lattice QCD, Phys. Rev. D **109**, 114514 (2024), arXiv:2402.09362 [hep-lat].

[36] M. Horoi and A. Neacsu, Shell model predictions for ^{124}Sn double-β decay, Phys. Rev. C **93**, 024308 (2016), arXiv:1511.03711 [nucl-th].

[37] Y. Iwata, N. Shimizu, T. Otsuka, Y. Utsuno, J. Menéndez, M. Honma, and T. Abe, Large-scale shell-model analysis of the neutrinoless $\beta\beta$ decay of ^{48}Ca, Phys. Rev. Lett. **116**, 112502 (2016), [Erratum: Phys.Rev.Lett. 117, 179902 (2016)], arXiv:1602.07822 [nucl-th].

[38] L. Coraggio, N. Itaco, G. De Gregorio, A. Gargano, R. Mancino, and S. Pastore, Present Status of Nuclear Shell-Model Calculations of $0\nu\beta\beta$ Decay Matrix Elements, Universe **6**, 233 (2020), arXiv:2011.14734 [nucl-th].

[39] L. Jokiniemi, P. Soriano, and J. Menéndez, Impact of the leading-order short-range nuclear matrix element on the neutrinoless double-beta decay of medium-mass and heavy nuclei, Phys. Lett. B **823**, 136720 (2021), arXiv:2107.13354 [nucl-th].

[40] L. Jokiniemi, B. Romeo, P. Soriano, and J. Menéndez, Neutrinoless $\beta\beta$-decay nuclear matrix elements from two-neutrino $\beta\beta$-decay data, Phys. Rev. C **107**, 044305 (2023), arXiv:2207.05108 [nucl-th].

[41] D. B. Kaplan, M. J. Savage, and M. B. Wise, Nucleon - nucleon scattering from effective field theory, Nucl. Phys. B **478**, 629 (1996), arXiv:nucl-th/9605002.

[42] D. B. Kaplan, M. J. Savage, and M. B. Wise, Two nucleon systems from effective field theory, Nucl. Phys. B **534**, 329 (1998), arXiv:nucl-th/9802075.

[43] D. B. Kaplan, M. J. Savage, and M. B. Wise, A New expansion for nucleon-nucleon interactions, Phys. Lett. B **424**, 390 (1998), arXiv:nucl-th/9801034.

[44] R. C. Farrell, I. A. Chernyshev, S. J. M. Powell, N. A. Zemlevskiy, M. Illa, and M. J. Savage, Preparations for quantum simulations of quantum chromodynamics in 1+1 dimensions. II. Single-baryon β-decay in real time, Phys. Rev. D **107**, 054513 (2023), arXiv:2209.10781 [quant-ph].

[45] R. C. Farrell, N. A. Zemlevskiy, M. Illa, and J. Preskill, Digital quantum simulations of scattering in quantum field theories using W states (2025), arXiv:2505.03111 [quant-ph].
[46] The CUDA-Q development team, CUDA-Q 0.8.0 (2024).
[47] A. Javadi-Abhari *et al.*, Quantum computing with Qiskit (2024), arXiv:2405.08810 [quant-ph].
[48] J. B. Kogut and L. Susskind, Hamiltonian Formulation of Wilson's Lattice Gauge Theories, Phys. Rev. D **11**, 395 (1975).
[49] T. Banks, L. Susskind, and J. B. Kogut, Strong Coupling Calculations of Lattice Gauge Theories: (1+1)-Dimensional Exercises, Phys. Rev. D **13**, 1043 (1976).
[50] R. Dempsey, I. R. Klebanov, S. S. Pufu, and B. Zan, Discrete chiral symmetry and mass shift in the lattice Hamiltonian approach to the Schwinger model, Phys. Rev. Res. **4**, 043133 (2022), arXiv:2206.05308 [hep-th].
[51] N. Klco, E. F. Dumitrescu, A. J. McCaskey, T. D. Morris, R. C. Pooser, M. Sanz, E. Solano, P. Lougovski, and M. J. Savage, Quantum-classical computation of Schwinger model dynamics using quantum computers, Phys. Rev. A **98**, 032331 (2018), arXiv:1803.03326 [quant-ph].
[52] T. V. Zache, N. Mueller, J. T. Schneider, F. Jendrzejewski, J. Berges, and P. Hauke, Dynamical Topological Transitions in the Massive Schwinger Model with a θ Term, Phys. Rev. Lett. **122**, 050403 (2019), arXiv:1808.07885 [quant-ph].
[53] C. Nagele, J. E. Cejudo, T. Byrnes, and M. Kleban, Flux unwinding in the lattice Schwinger model, Phys. Rev. D **99**, 094501 (2019), arXiv:1811.03096 [hep-th].
[54] Z. Davoudi, C.-C. Hsieh, and S. V. Kadam, Quantum computation of hadron scattering in a lattice gauge theory (2025), arXiv:2505.20408 [quant-ph].
[55] Y. Chai, Y. Guo, and S. Kühn, Towards Quantum Simulation of Meson Scattering in a Z2 Lattice Gauge Theory (2025), arXiv:2505.21240 [quant-ph].
[56] R. C. Farrell, M. Illa, A. N. Ciavarella, and M. J. Savage, Quantum simulations of hadron dynamics in the Schwinger model using 112 qubits, Phys. Rev. D **109**, 114510 (2024), arXiv:2401.08044 [quant-ph].
[57] B. Efron, Bootstrap Methods: Another Look at the Jackknife, Ann. Statist. **7**, 1 (1979).
[58] D. M. Debroy and K. R. Brown, Extended flag gadgets for low-overhead circuit verification, Phys. Rev. A **102**, 052409 (2020).
[59] J. M. Arrazola, O. Di Matteo, N. Quesada, S. Jahangiri, A. Delgado, and N. Killoran, Universal quantum circuits for quantum chemistry, Quantum **6**, 742 (2022).
[60] J. Goings, L. Zhao, J. Jakowski, T. Morris, and R. Pooser, Molecular symmetry in vqe: A dual approach for trapped-ion simulations of benzene, in *2023 IEEE International Conference on Quantum Computing and Engineering (QCE)* (IEEE, 2023) p. 76–82.
[61] R. T. Sutherland, Quantum logic for state preparation, readout, and leakage detection with binary subspace measurements, Phys. Rev. A **111**, 052627 (2025), arXiv:2410.23531 [quant-ph].
[62] Y.-H. Chen and C. H. Baldwin, Randomized Benchmarking with Leakage Errors (2025), arXiv:2502.00154 [quant-ph].
[63] R. Stricker, D. Vodola, A. Erhard, L. Postler, M. Meth, M. Ringbauer, P. Schindler, T. Monz, M. Müller, and R. Blatt, Experimental deterministic correction of qubit loss, Nature **585**, 207–210 (2020).
[64] S. R. Beane, W. Detmold, K. Orginos, and M. J. Savage, Uncertainty Quantification in Lattice QCD Calculations for Nuclear Physics, J. Phys. G **42**, 034022 (2015), arXiv:1410.2937 [nucl-th].
[65] B. Hall, A. Roggero, A. Baroni, and J. Carlson, Simulation of collective neutrino oscillations on a quantum computer, Phys. Rev. D **104**, 063009 (2021), arXiv:2102.12556 [quant-ph].
[66] K. Yeter-Aydeniz, S. Bangar, G. Siopsis, and R. C. Pooser, Collective neutrino oscillations on a quantum computer, Quant. Inf. Proc. **21**, 84 (2022), arXiv:2104.03273 [quant-ph].
[67] M. Illa and M. J. Savage, Basic elements for simulations of standard-model physics with quantum annealers: Multigrid and clock states, Phys. Rev. A **106**, 052605 (2022), arXiv:2202.12340 [quant-ph].
[68] V. Amitrano, A. Roggero, P. Luchi, F. Turro, L. Vespucci, and F. Pederiva, Trapped-ion quantum simulation of collective neutrino oscillations, Phys. Rev. D **107**, 023007 (2023), arXiv:2207.03189 [quant-ph].
[69] M. Illa and M. J. Savage, Multi-Neutrino Entanglement and Correlations in Dense Neutrino Systems, Phys. Rev. Lett. **130**, 221003 (2023), arXiv:2210.08656 [nucl-th].
[70] P. Siwach, K. Harrison, and A. B. Balantekin, Collective neutrino oscillations on a quantum computer with hybrid quantum-classical algorithm, Phys. Rev. D **108**, 083039 (2023), arXiv:2308.09123 [quant-ph].
[71] F. Turro, I. A. Chernyshev, R. Bhaskar, and M. Illa, Qutrit and qubit circuits for three-flavor collective neutrino oscillations, Phys. Rev. D **111**, 043038 (2025), arXiv:2407.13914 [quant-ph].
[72] L. Spagnoli *et al.*, Collective neutrino oscillations in three flavors on qubit and qutrit processors, Phys. Rev. D **111**, 103054 (2025), arXiv:2503.00607 [quant-ph].
[73] A. Maksymov, J. Nguyen, Y. Nam, and I. Markov, Enhancing quantum computer performance via symmetrization (2023), arXiv:2301.07233 [quant-ph].
[74] Z. Jiang, K. J. Sung, K. Kechedzhi, V. N. Smelyanskiy, and S. Boixo, Quantum algorithms to simulate many-body physics of correlated fermions, Phys. Rev. Applied **9**, 044036 (2018), arXiv:1711.05395 [quant-ph].
[75] D. Wecker, M. B. Hastings, N. Wiebe, B. K. Clark, C. Nayak, and M. Troyer, Solving strongly correlated electron models on a quantum computer, Phys. Rev. A **92**, 062318 (2015).
[76] I. D. Kivlichan, J. McClean, N. Wiebe, C. Gidney, A. Aspuru-Guzik, G. K.-L. Chan, and R. Babbush, Quantum Simulation of Electronic Structure with Linear Depth and Connectivity, Phys. Rev. Lett. **120**, 110501 (2018).
[77] R. C. Farrell, M. Illa, A. N. Ciavarella, and M. J. Savage, Scalable Circuits for Preparing Ground States on Digital Quantum Computers: The Schwinger Model Vacuum on 100 Qubits, PRX Quantum **5**, 020315 (2024), arXiv:2308.04481 [quant-ph].

[78] H. R. Grimsley, S. E. Economou, E. Barnes, and N. J. Mayhall, An adaptive variational algorithm for exact molecular simulations on a quantum computer, Nat. Commun. **10**, 3007 (2019), arXiv:1812.11173 [quant-ph].
[79] R. C. Farrell, M. Illa, and M. J. Savage, Steps toward quantum simulations of hadronization and energy loss in dense matter, Phys. Rev. C **111**, 015202 (2025), arXiv:2405.06620 [quant-ph].
[80] M. G. Algaba, P. V. Sriluckshmy, M. Leib, and F. Šimkovic IV, Low-depth simulations of fermionic systems on square-grid quantum hardware, Quantum **8**, 1327 (2024), arXiv:2302.01862 [quant-ph].
[81] R. C. Farrell, I. A. Chernyshev, S. J. M. Powell, N. A. Zemlevskiy, M. Illa, and M. J. Savage, Preparations for quantum simulations of quantum chromodynamics in 1+1 dimensions. I. Axial gauge, Phys. Rev. D **107**, 054512 (2023), arXiv:2207.01731 [quant-ph].
[82] I. Chernyshev, *Developing techniques for Simulation of SU(3) Quantum Field Theories on State-of-the-Art Quantum Devices*, Ph.D. thesis, University of Washington (2025), arXiv:2502.02502 [quant-ph].
[83] A. Cervera-Lierta, Exact Ising model simulation on a quantum computer, Quantum **2**, 114 (2018), arXiv:1807.07112 [quant-ph].
[84] T. F. Stetina, A. Ciavarella, X. Li, and N. Wiebe, Simulating Effective QED on Quantum Computers, Quantum **6**, 622 (2022), arXiv:2101.00111 [quant-ph].
